# Conjugated C-6 hydroxylated bile acids in serum relate to human metabolic health and gut *Clostridia* species

**DOI:** 10.1038/s41598-021-91482-y

**Published:** 2021-06-24

**Authors:** Anders Ø Petersen, Hanna Julienne, Tuulia Hyötyläinen, Partho Sen, Yong Fan, Helle Krogh Pedersen, Sirkku Jäntti, Tue H. Hansen, Trine Nielsen, Torben Jørgensen, Torben Hansen, Pernille Neve Myers, H. Bjørn Nielsen, S. Dusko Ehrlich, Matej Orešič, Oluf Pedersen

**Affiliations:** 1 Department of Health Technology, Technical University of Denmark, 2800 Lyngby, Denmark; 2 Department of Computational Biology—USR 3756 CNRS, Institut Pasteur, 75015 Paris, France; 3 School of Science and Technology, Örebro University, Örebro, Sweden; 4 Turku Bioscience Centre, University of Turku and Åbo Akademi University, 20520 Turku, Finland; 5 Novo Nordisk Foundation Center for Basic Metabolic Research, Faculty of Health and Medical Sciences, University of Copenhagen, Blegdamsvej 3B, 2200 Copenhagen, Denmark; 6 Clinical Microbiomics A/S, Fruebjergvej 3 , 2100 Copenhagen, Denmark; 7 Drug Research Program, Division of Pharmaceutical Chemistry and Technology, Faculty of Pharmacy, University of Helsinki, 00014 Helsinki, Finland; 8 Department of Public Health, Faculty of Health and Medical Sciences , University of Copenhagen , Blegdamsvej 3B , 2200 Copenhagen, Denmark; 9 Faculty of Medicine, Aalborg University, Niels Jernes Vej 10, 9200 Aalborg East, Denmark; 10 School of Medical Sciences, Örebro University, 702 81 Örebro, Sweden

**Keywords:** Obesity, Microbiota

## Abstract

Knowledge about in vivo effects of human circulating C-6 hydroxylated bile acids (BAs), also called muricholic acids, is sparse. It is unsettled if the gut microbiome might contribute to their biosynthesis. Here, we measured a range of serum BAs and related them to markers of human metabolic health and the gut microbiome. We examined 283 non-obese and obese Danish adults from the MetaHit study. Fasting concentrations of serum BAs were quantified using ultra-performance liquid chromatography-tandem mass-spectrometry. The gut microbiome was characterized with shotgun metagenomic sequencing and genome-scale metabolic modeling. We find that tauro- and glycohyocholic acid correlated inversely with body mass index (*P* = 4.1e-03, *P* = 1.9e-05, respectively), waist circumference (*P* = 0.017, *P* = 1.1e-04, respectively), body fat percentage (*P* = 2.5e-03, *P* = 2.3e-06, respectively), insulin resistance (*P* = 0.051, *P* = 4.6e-4, respectively), fasting concentrations of triglycerides (*P* = 0.06, *P* = 9.2e-4, respectively) and leptin (*P* = 0.067, *P* = 9.2e-4). Tauro- and glycohyocholic acids, and tauro-a-muricholic acid were directly linked with a distinct gut microbial community primarily composed of *Clostridia* species (*P* = 0.037, *P* = 0.013, *P* = 0.027, respectively). We conclude that serum conjugated C-6-hydroxylated BAs associate with measures of human metabolic health and gut communities of *Clostridia* species. The findings merit preclinical interventions and human feasibility studies to explore the therapeutic potential of these BAs in obesity and type 2 diabetes.

## Introduction

Bile acids (BAs) are a class of steroids produced from cholesterol in the liver where they are conjugated with either glycine or taurine and released into the bile. These primary BAs, which in humans predominantly comprise of cholic acid (CA) and chenodeoxycholic acid (CDCA) are absorbed by different intestinal bacteria, and conjugates of glycine or taurine are removed with subsequent alteration of hydroxyl groups to form secondary BAs^1^. BAs emulsify dietary fats in the small intestine and facilitate their absorption^1,2^. At the terminal ileum, the majority of BAs are absorbed by the apical sodium dependent BA transporters and enter the hepatic portal circulation. In total, about 95% of BAs are reabsorbed into intestinal enterocytes, whilst > 5% of BAs enter the systemic circulation and act on multiple organs throughout the body via several types of receptors^3^. The most prominent of these are the farnesoid X receptor (FXR) and the Takeda G protein-coupled membrane receptor 5 (TGR5) which are expressed in a variety of peripheral tissues^4–7^. Through these receptors, BAs regulate biological processes such as immunity, neuroprotection, metabolism, and energy expenditure^8–12^.

At present, we have sparse knowledge about the biosynthesis and the functions of a considerable part of the human blood profile of BAs due to the biological differences between human and commonly used animal models like pigs and rodents^13,14^. A large part of primary BAs in pigs and rodents consists of C-6 hydroxylated hyocholic acid (HCA) and hyodeoxycholic acid (HDCA) and their conjugated derivatives, also called γ-muricholic acid^14,15^. In pigs, these C-6 hydroxylated BAs may comprise about 5% of the circulating BA pool and there is growing evidence that these specific BAs may contribute to maintain glucose homeostasis in domesticated pigs that are resistant to development of type 2 diabetes despite living in an obesogenic environment^14,15^. The dominating BAs in humans are different. Here, the primary BAs consist of cholic acids (CA) and chenodeoxycholic acids (CDCA), which are not hydroxylated at the C-6 position^15^.

Recent studies have demonstrated, however, a previously underappreciated diversity of BAs in human blood including small amounts of HCA and HDCA and their derivatives^13^. Here, we tested two related hypotheses, (i) a variety of C-6 hydroxylated BAs are measurable in human serum and are associated with human metabolic health, and (ii) the serum concentration of these C-6-hydroxylated BAs are associated with distinct co-abundant microbial species in the human gut microbiota.

## Materials and methods

### Study participants

Study participants were recruited from the Danish Inter99 study and were originally selected for the MetaHit study on microbiota gene richness^16^. In the present study, we measured serum BAs in 283 Danes of whom 106 were non-obese (BMI < 27 kg/m^2^) and 177 were obese (BMI > 27 kg/m^2^). None of the volunteers had undergone bariatric surgery, and none had taken antibiotics two months prior to their inclusion in the study protocol. Median age of the study population was 56.4 ± (standard deviation (SD) = 7.3) years, mean BMI was (29.5 ± 5.99 kg/m^2^); 133 were male and 150 were female. None of the participants were diagnosed with diabetes mellites (Type 1 or 2). See electronic supplementary notes (ESM) for gender-stratified statistics. 34 of the study participants were taking statins at the time of study. We compared the BA concentrations between the group of subjects with or without administration of statins by Mann–Whitney U test, and found no significant differences (*P* < 0.05).

Details of clinical and biochemical phenotyping, stool sampling and microbial DNA purification, library preparation, Illumina shotgun sequencing of microbial DNA and quality assurance of sequencing reads have been reported^16^. The study was approved by the local Ethical Committees of the Capital Region of Denmark (HC-2008-017), and was in accordance with the principals of the Declaration of Helsinki. All individuals gave written informed consent before participation in the study.

### Measurement of bile acids in fasting serum

Blood samples were obtained in the morning after a minimum 10 h of overnight fasting. BA levels were measured in serum by ultra-performance liquid chromatography-tandem mass-spectrometry (UPLC-QqQMS)^17^. Details on sample preparation and molecular analysis of BAs are given in (ESM).

### Gut bacterial abundance and bacterial gene count

Illumina reads were mapped to the Integrated Gene Catalogue version 9.9 to construct a gene count matrix^18^. Reads that mapped onto several genes were attributed with the smart shared read method. The read count matrix was then downsized to 8 M reads. Gene abundances were normalized by the gene length and the total number of reads (Reads Per Kilobase Per Million mapped reads (RPKM) normalization).

### Construction of metagenomic species and taxonomic annotation

The gene count matrix was used to cluster metagenomic species (MGS) based on co-occurrence by use of the canopy clustering software developed by Nielsen *et. al*^19^. We assembled 3,463 co-abundant Cluster Genes (CAG) having 50 genes or more. In the present study, 1,437 CAGs were composed of more than 500 genes and were considered as MGS. Taxonomic annotation of MGS was based on the aggregation of its gene annotation applying *‘BLASTn’* as previously reported^19^.

We computed MGS abundance using the *‘momr’* R package (https://cran.r-project.org/web/packages/momr/index.html). MGS abundance was defined as the mean abundance of the 50 MGS genes that correlated strongest to the median gene abundance. We considered the MGS abundance in a sample to be null, if fewer than 10% of its genes were present.

### Graph inference

We implemented the Gleesso (https://github.com/hjulienne/Gleesso_Pipeline) pipeline to adapt the SPIEC-EASI pipeline to shotgun MGS abundances and to construct gut bacterial communities. A detailed step-by-step description of the applied community construction approach is given in (ESM).

### Construction of robust gut bacterial communities

To assess the robustness of the gut community composition detected by the Gleesso pipeline we applied the following: (1) The Gleesso pipeline to 100 different subsets of 80% of the study sample stratified for bacterial gene richness group^16^, i.e., 170 samples with high bacterial gene richness and 57 samples with low bacterial gene richness). (2) Only species assigned to the same community on more than 60% of subsets were included in the distal analyses. Abundance was computed on the robust community composition. We defined the stability index of a species as the fraction of the subset where it was assigned to the same community. Details about the correlation analysis are given in (**ESM**).

### Logistic ridge regression analyses of serum bile acids in relation to measures of adiposity and metabolism

We constructed logistic ridge regressions with the python ‘*scikit-learn’* library: (http://scikit-learn.org/stable/modules/linear_model.html#ridge-regression). Serum levels of BAs were scaled prior to model fitting, so the coefficients would be comparable to one another. The target variables were BMI, HOMA-IR and fasting serum levels of leptin. The latter variables were binarized around their median values. The BMI target was binarized around 27 kg/m^2^. Models were fitted separately for women and men. We drew 10,000 partitions of the study sample by randomly splitting the 80% of study samples in a training set and 20% in a test set. For more detail, please see (ESM).

### Genome-scale metabolic modeling of bile acid biosynthesis

We applied genome-scale metabolic modeling (GSMM) of gut microbiota to achieve insights into the BA biosynthetic and biotransformation potentials / pathway(s). MGS with more than 500 genes were listed and mapped to the AGORA (version 1.02) compendium, comprising 773 gut bacteria (including 205 genera and 605 species)^20^. AGORA is a comprehensive resource of semi-automatically reconstructed GEMs for the human gut microbiota. A detailed methodology of GSMM applied to BA metabolism can be found in the (ESM). We extended the GSMM framework by adding a common compartment simulating the gut epithelial cells (Supplementary Dataset 1), and another compartment which represented the host body fluids, and finally a common gut lumen compartment that enabled the gut bacteria and the host epithelial cells to exchange dietary metabolites and BAs.

In the intestinal epithelial cells, some BA transporters (for e.g. ABC transporter) have been previously identified^21^. Transporters for HCA, particularly THCA (THCA–ABC BA transporter, bicarbonate counter transporter and sodium cotransporter) have been identified and reported in Recon3D^22^, a generic human metabolic reconstruction. The BA transporters obtained from Recon3D and several other pieces of bibliographic evidence were added to the gut epithelial cell model developed in this study (Supplementary Dataset 1)^8,12,23,24^. A report on the AGORA approach deployed in the study can be found in the (ESM).

### Search for clusters of gut bacterial genes involved in biotransformation of bile acids

We attempted to identify intestinal bacterial genes with the potential to produce C-6 hydroxylated BAs. We collapsed 2,250,421 microbial genes into 3,812 Clusters of Orthologous Groups (COGs) of proteins. We hypothesized that structurally similar proteins carry similar functions, and certain COGs could confer the ability to modify certain BAs. We grouped the BAs by their hydroxylation patterns, irrespective of their conjugation type(s) for a specific BA group (BAG), e.g. by collapsing GCDCA, TCDCA and CDCA into CDCAs. The concentration of a BAG was computed as the sum of the constituent BA concentrations. Pairs of BAGs that differed in only one hydroxylation position by presence/absence of steric orientation were identified, and ratios between the concentrations of these BAGs were computed for each individual. Next, these ratios were correlated with each MGS by using Spearman’s correlation analyses. Finally, by applying a Wilcoxon test, we compared the correlation strengths between the MGSs that did or did not contain a given COG. Associated COGs were deemed to be positively or inversely associated with a BAG ratio of interest based on the median correlation coefficient of the MGS that contained the COG. Further, these associated COGs for each BAG ratio were mapped onto *STRING v10.5*, a resource that curates the relatedness of COGs and proteins^25^. Connections with one intermediary COG was allowed, in which case the sum product of connection strengths was evaluated as the connectivity.

Additionally, a χ^2^ test was employed to determine if there was any significant degree of dependency between MGS correlation to a BAG ratio of interest and the presence of a COG. BAG was correlated by Spearman’s correlation to each BAG and correlation strengths were saved. For each COG, we identified which MGSs did and did not contain a gene corresponding to that given COG. In order to investigate whether the presence of a given COG conferred the ability to shift the ratio of BAGs, MGSs were indexed on two conditions; whether or not they contained a given COG and whether they correlated positively or inversely with a given BAG ratio of interest. A χ^2^ test was employed to determine dependency between these two traits.

### Study flow

**Figure 1:**
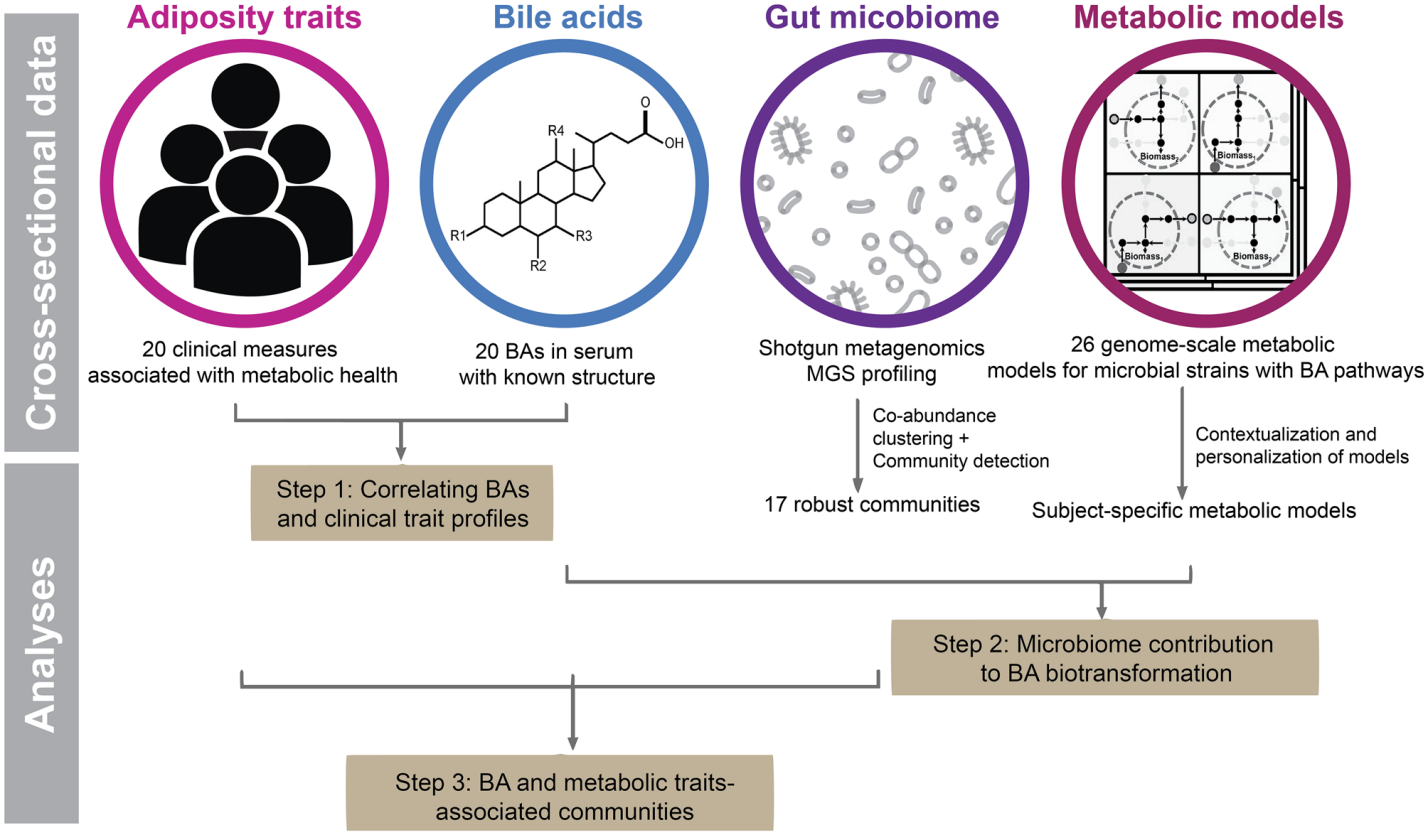
Framework for analyses of three complementary data sets: adiposity/metabolic traits, fasting serum bile acids and gut metagenomics species including search for bacterial enzymes or pathways involved in biotransformation of bile acids. The analysis addresses three main questions; (i) how does the serum bile acid composition relate to markers of host metabolic health? (ii) search for microbial pathways involved in bile acids biosynthesis. Is there evidence for certain gut bacterial species to harbor a genetic potential for C-6 hydroxylated bile acid biosynthesis or their uptake from the gut to the systemic circulation? (iii) what is the relationship between gut microbiota composition (as described by communities of co-abundant species), fasting serum bile acids and metabolic phenotypes?

Figure 1 outlines the analytical study flow. In step 1, we associated fasting serum concentrations of BAs with the bio-clinical traits of study participants focusing on the measures of adiposity and metabolism. In step 2, we associated the BA-related phenotypes with the gut bacterial species. In step 3, we performed GSMM of gut microbiota to achieve a putative mechanistic understanding about the regulation of BA biotransformation pathways in obese and non-obese individuals. Supplementary Table S1 lists the clinical characteristics of 283 study participants including measures of body composition and markers of metabolism and immunity.

## Results

### Bile acids in fasting human serum

**Figure 2:**
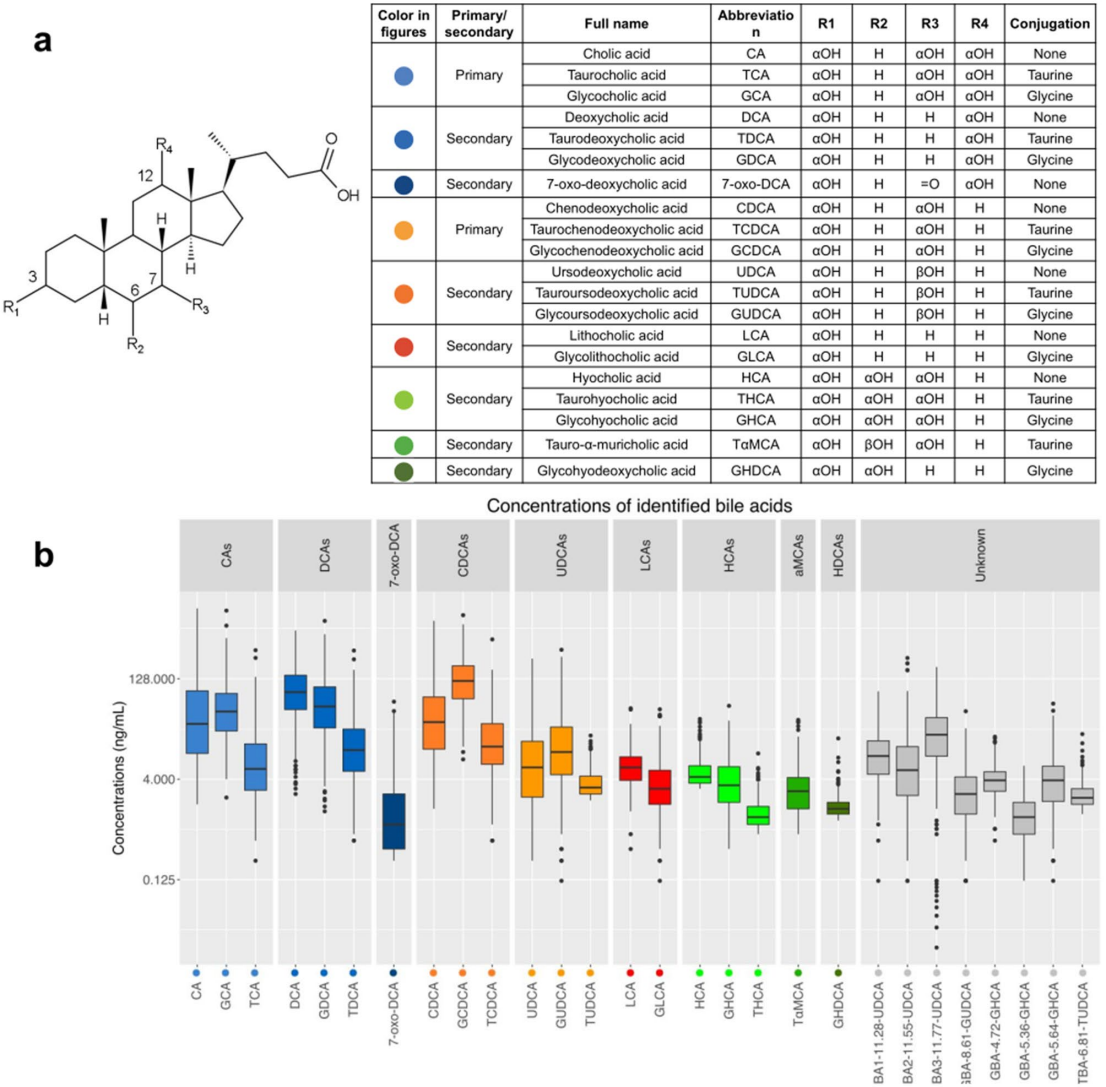
Chemical structures of bile acids and their concentrations in human fasting serum. (**a**) Summary of the structures and abbreviations of 20 serum bile acids with known chemical structure measured in the present study. We refer to the structure above for the positions of radical groups R1-4. The ‘αOH’ refers to an alpha-hydroxyl group pointing away from the reader, ‘βOH’ refers to a beta-hydroxyl group pointing towards the reader, ‘=O’ refers to a double bonded oxygen, ‘H’ refers to a hydrogen. Number refers to the number of a given carbon atom. Color-coding is chosen based on shared structural traits. Blue colors are for cholic acid (CA) and its metabolites with a R4 hydroxyl-group; red and orange colors are for chenodeoxycholic acid (CDCA) and conjugates with hydroxylations at R3 but not R4 position. Green colors are used for compounds with a hydroxyl group at R2 position. (**b**) An overview of the fasting serum concentrations of 20 bile acids of known chemical structures, and eight bile acids of ‘unknown’ structures from all the study participants. Abbreviations and bile acid names are shown in (**a**).

Serum BAs present in at least 50% of the study participants were considered for the data analysis. 20 BAs with known chemical structure and eight with unresolved stereochemistry structures were included in the final dataset (Fig. 2a). While exact stereochemistry is unknown, hydroxylation and conjugation can be inferred. Serum concentrations of BAs were detected in the range of 0.125—332.0 ng/ml (Fig. 2B). Of the 28 BAs, eight were C-6 hydroxylated BAs. Five of these with known stereochemistry: HCA, GHCA, THCA, TαMCA, GHDCA, and three of unknown stereochemistry (Supplementary Fig. S1).

### C-6 hydroxylated bile acids associated with markers of adiposity and metabolism

**Figure 3:**
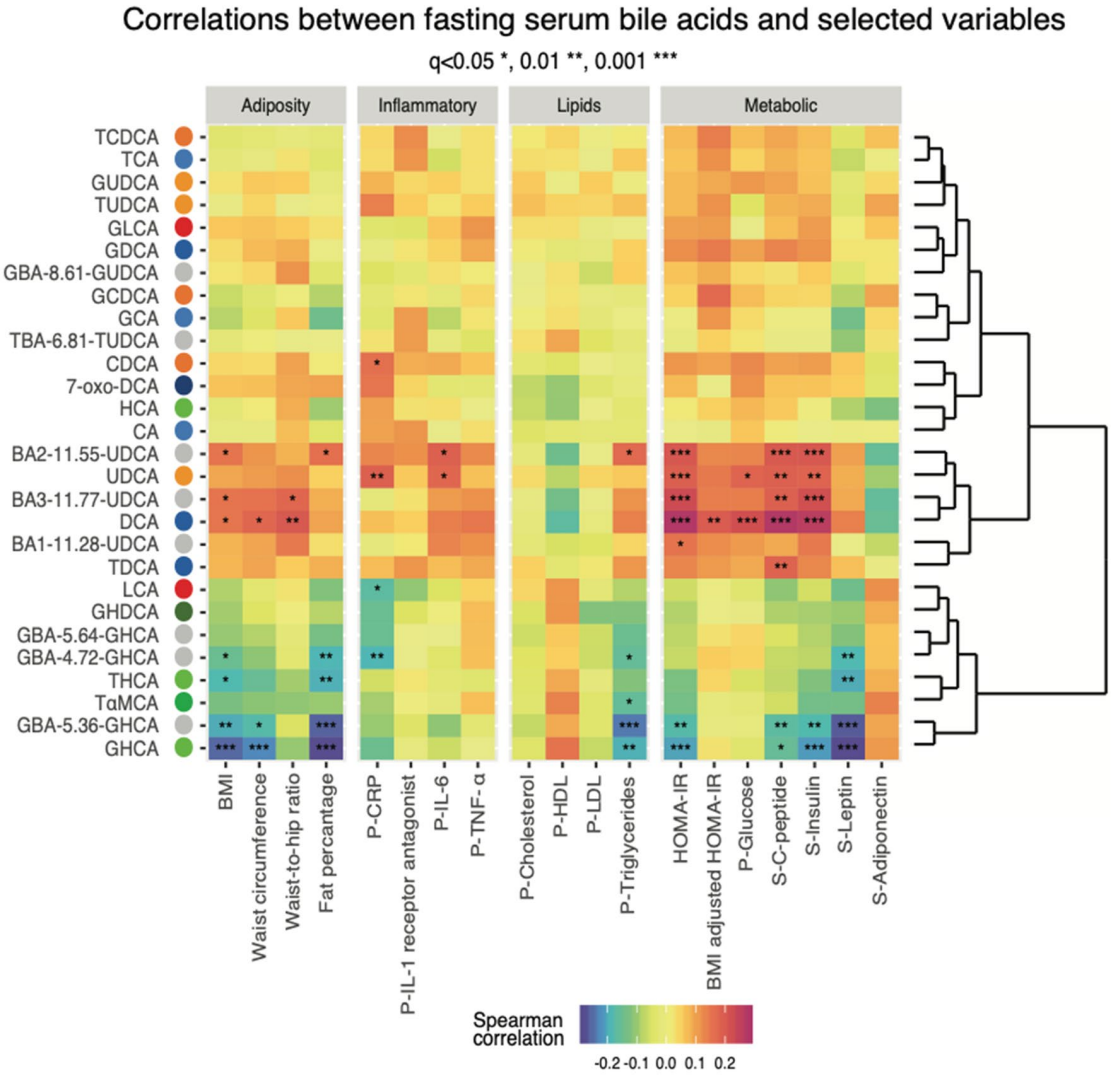
Correlations between fasting serum bile acids and bio-clinical traits in the study population of 283 individuals. Heatmap showing outcomes of Spearman’s correlation analyses between measured fasting serum concentrations of bile acids and adiposity measures and metabolic traits. P-values are corrected by the Benjamini-Hochberg (FDR) approach. The level of significance is indicated by stars in each square. *P < 0.05, **P < 0.025, ***P < 0.01. See (**a**) for the correspondence between the abbreviated bile acid names and the complete bile acid structure. HOMA-IR was adjusted for BMI by a linear model, and the residuals were used as HOMA-IR adjusted for BMI. Abbreviated names preceded with G or T indicate that the bile acid is conjugated with glycine or taurine. *CRP* C-reactive protein, *IL* interleukin, *LDL* low density lipoprotein, *HDL* high density lipoprotein, *TNF* tumor necrosis factor, prefix P- and S- indicate plasma and serum, respectively.

In 283 individuals, the serum concentrations of several conjugated C-6 hydroxylated BAs correlated inversely with a range of adiposity markers. GHCA was inversely associated (Spearman’s correlation, FDR-corrected *P* < 0.05) with BMI, waist circumference, body fat percentage, insulin resistance (HOMA-IR), fasting circulating levels of triglycerides and leptin (*P* = 5.3e-4, 3.1e-3, 6.7e-5, 3.8e-3, 1.3e-2, 5.1e-5, respectively, FDR-corrected). THCA was inversely correlated with BMI, body fat percentage, and leptin (*P* = 0.04, 0.017, 0.014, respectively, FDR-corrected) (Fig. 3).

In contrast, non-conjugated HCA was not significantly associated with any of commonly utilized measures of adiposity (*P* > 0.05, Spearman’s correlation) (Fig. 3). TαMCA, a stereoisomer of THCA through epimerization of the hydroxyl group at C-6 was inversely associated with fasting serum triglycerides (*P* = 7.07e-3, Spearman’s correlation). Despite only being significantly correlated with one adiposity trait, this BA clustered with THCA and GHCA for their similar correlations. In addition, several conjugated C-6 hydroxylated BAs with unresolved stereochemistry (GBA-5.36-GHCA, GBA-5.64-GHCA and GBA-4.72-GHCA) were inversely associated with adiposity traits, similar to associations observed with THCA and GHCA. By contrast, serum concentrations of DCA, UDCA, and their conjugates were positively associated with fasting circulating levels of glucose, insulin and C-peptide, adiposity measures and HOMA-IR (Fig. 3).

Next, we applied Logistic Ridge Regression (LR) modeling to assess the impact of various BA concentrations on key indicators of metabolic health: BMI, HOMA-IR and fasting serum concentration of leptin. We binarized targets around the median for HOMA-IR and serum concentrations of leptin, while BMI was binarized around 27 kg/m^2^ (obese vs. non-obese, Supplementary Fig. S2). To account for the sex-specific differences in the BA concentration, the LR models were applied separately for men and women (Supplementary Table S1).

As shown in (Supplementary Table 2), areas under the curve (AUC) of the LR models for both BMI and HOMA-IR were significantly above the null model (AUC = 0.5), suggesting that fasting serum levels of BAs were able to distinguish between high and low values of BMI and HOMA-IR. Despite some differences in BA metabolism between men and women, most BAs exhibited similar associations among sexes, as demonstrated by the LR model coefficients. The serum concentration of THCA was inversely associated with obesity and related metabolic traits for both men and women. Higher levels of serum THCA were linked with a 17% lower prevalence of obesity for women, and 14% for men (Supplementary Fig. S3). Intriguingly, CDCA and UDCA were associated with an increase in odds of having high HOMA-IR for women, but not for men (Supplementary Fig. S4). Additionally, higher levels of serum DCA was associated with a 22% higher prevalence of obesity in men and 9.4% in women (Supplementary Fig. S3 and S4). The findings may suggest sex-specific effects of BAs.

### Communities of gut bacterial species were linked with serum bile acids

**Figure 4:**
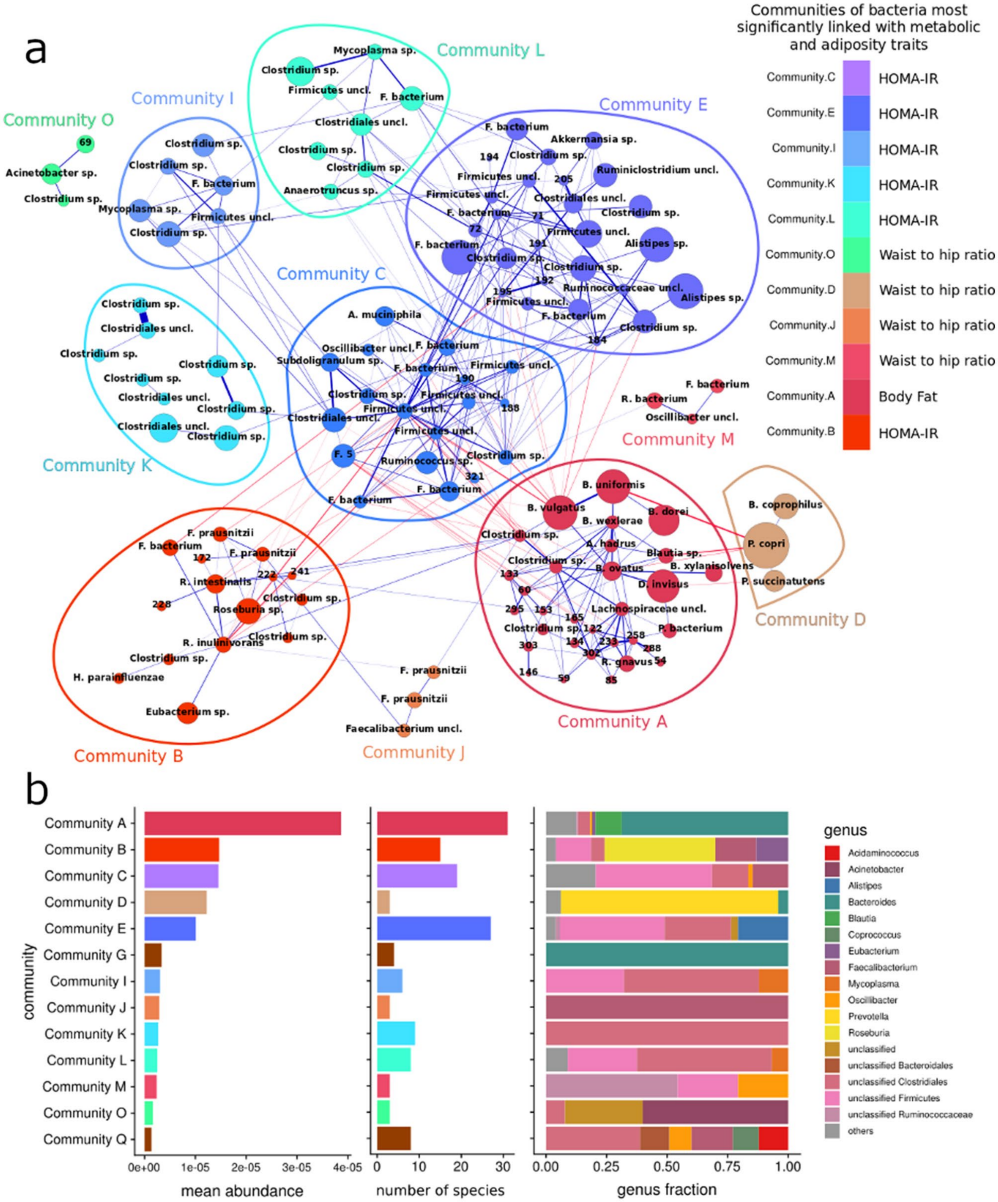
Co-occurrence networks of gut bacteria identified in metagenomics analyses of 283 middle-aged adults and their relationships with adiposity measures and metabolic traits. (**a**) Co-occurrence networks of species (MGSs) in communities associated with adiposity measures and metabolic phenotypes. Node size reflects mean abundance of species across samples (see Supplementary Dataset 2). Node colors corresponds to the community attribution. Blue and red color edges represent positive and inverse correlation coefficients respectively. Thickness of an edge is proportional to the absolute value of the correlation coefficient (see Methods and Supplementary Dataset 2 for exact value). (**b**) Microbial community content: mean abundance across all samples (left panel); number of species in each community (middle panel); and the relative abundance of genera in the communities is given in the right panel.

**Figure 5:**
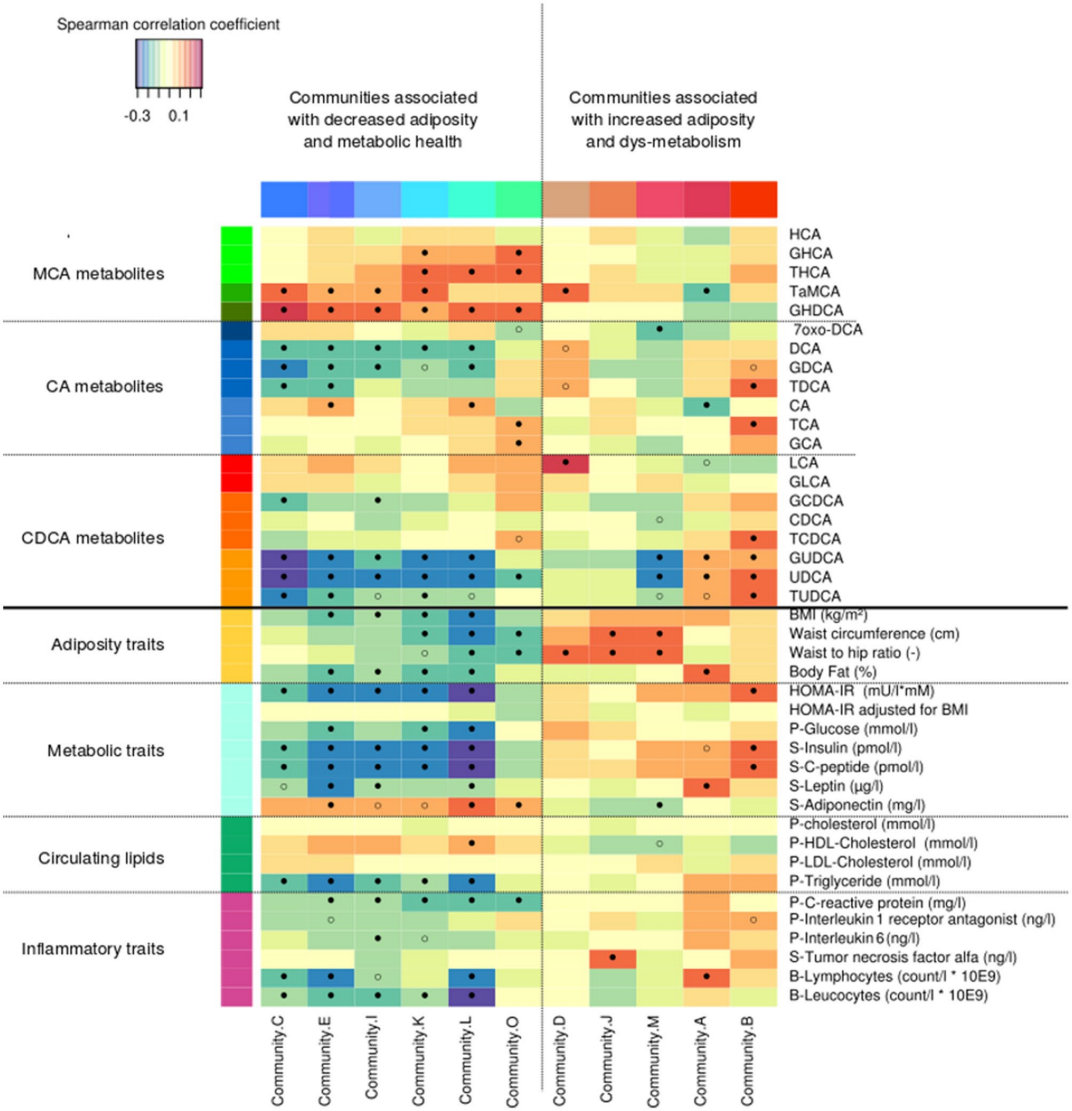
Interplay of the intestinal microbiota, fasting serum levels of bile acids and markers of metabolic health in 283 individuals. Heatmap depicting Spearman’s correlations between microbial communities’ abundances and serum bile acids (top) or bio-clinical traits (bottom), respectively. The level of significance is indicated by the point inside squares: empty circles indicate *P* < 0.05; filled circles indicate q < 0.05. Only gut microbial communities with at least one significant correlation (*P* < 0.05) are shown. Prefix P- and S- indicate plasma and serum, respectively. See (**a**) for the correspondence between the abbreviated bile acid names.

One hundred and eighty-nine of the most abundant (1463) MGSs were organized into 17 bacterial communities (see Methods, ESM and Supplementary Dataset 2). The combined abundance of the species in these communities represented 47% of total gut bacterial abundance. Communities differed in number of species and their abundances (Fig. 4b), and were named A to Q, and ordered by decreasing mean abundance within a community. In general, the communities tended to include species of the same genus (Fig. 4b), possibly reflecting adaptation of similar taxa to a similar intestinal ecology. Distinct genera dominated the four most abundant bacterial communities. In communities where no consistent genus-level annotation could be attributed to the majority of members, the lowest resolution taxonomy was applied; e.g., *Bacteroides*, *Roseburia*, unclassified *Firmicutes*, and *Prevotella*. We assessed association between species communities and the serum BA pool composition by computing Spearman’s rank correlation coefficients (ρ) between abundance of bacterial communities and fasting serum concentration of BAs (Fig. 5). We found 11 of the 17 species communities that were associated with traits of metabolism and adiposity measures (Fig. 4a).

Community K, which was composed of *Clostridia spp.* (Fig. 4a)*,* correlated positively with the serum levels of C-6 hydroxylated GHCA, THCA, GHDCA and TαMCA with FDR-corrected p-values of 0.037, 0.013, 0.041 and 0.027, respectively. The serum concentration of TαMCA correlated positively with the C, E, I species communities. By contrast, community A correlated inversely with the circulating C-6 hydroxylated BAs. The communities C, E, I, and L, comprising chiefly *Firmicutes* and *Clostridiales spp.,* correlated inversely with the serum levels of the C-12 hydroxylated UDCA and its conjugates, but positively with the serum concentrations of C-6 hydroxylated BAs. Community B associated positively with serum concentrations of taurine conjugated BAs TDCA, TUDCA, TCDCA, TCA, with FDR-corrected p-values of 0.0092, 0.015, 0.01 and 0.023, respectively (Fig. 5). The abundance of this community correlated directly with the sum of the abundances of all the taurine conjugated BAs (ρ = 0.16, *P* = 5e-3, nominal). The finding may indicate that the species of this community benefit from a gut ecology rich in sulfur.

Several bacterial communities were inversely associated with the ratio of secondary to primary BAs (the sum of the concentrations of CA, CDCA and their conjugates, divided by the concentration of all other BAs with elucidated structures), namely communities E, K and L (ρ = -0.15, -0.12, -0.16, nominal *P* = 0.012, 0.04, 0.0056, respectively, not shown in Fig. 5).

In summary, these results suggest that secondary BAs in circulation are influenced by numerous gut bacterial species with a correspondingly complex patchwork of yet non-annotated bacterial genes and derived metabolic pathways.

### Genome-scale modeling failed to identify gut bacterial pathways related to biosynthesis of C-6 hydroxylated bile acids

**Figure 6:**
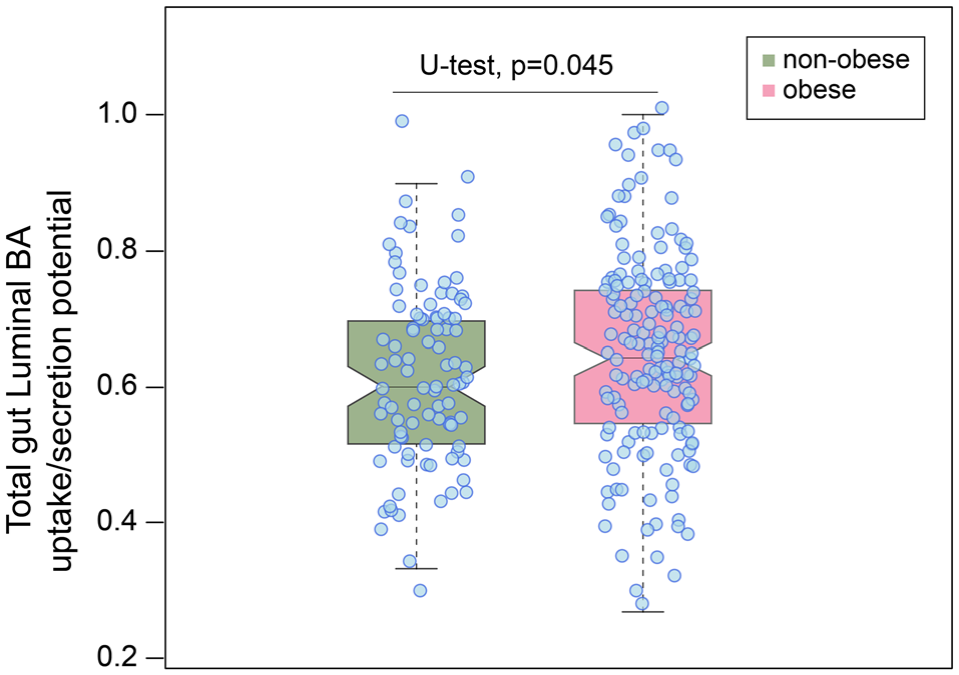
Modeling of the intestinal luminal bile acids uptake/secretion potentials in non-obese and obese individuals. Box plot showing a significant difference in the total luminal bile acid potentials for bile acid uptake/secretion between non-obese (n = 106) and obese (n = 177) subjects as evaluated by Mann–Whitney U test. The statistical significance is given by (*P* < 0.05).

To identify dysregulation of microbial pathways related to BA biosynthesis and transformation in the obese (n = 177) vs. non-obese (n = 106) individuals (BMI binarized around 27 kg/m^2^), we applied GSMM. We identified 26 abundant microbial species (see Methods) that have metabolic reconstructions or genome-scale metabolic models (GEMs) in the AGORA compendium (Supplementary Fig. S5 and S6). These GEMs were primarily enriched with bile salt hydrolysis reactions carried out by the bile salt hydrolase (BSH) enzyme (Supplementary Fig. S7). By using abundances of the microbes in each subject, we contextualized and personalized these microbial GEMs for obese and non-obese individuals (see Methods). GSMM of BA metabolism suggested that the luminal BA reactions, i.e., genes encoding BA reactions/pathways in both obese and non-obese individuals were active (Supplementary Fig. S7). Intriguingly, the metabolic potentials of the BA pathways were different between these groups. The total estimated metabolic potential (EMP) of the BA exchange reactions in the gut lumen was elevated in the obese *vs.* non-obese groups (*P* = 0.045, U-Test, Fig. 6). This finding is in line with the elevated serum concentration of CDCA, UDCA and DCA in the obese individuals (Supplementary Fig. S5 and Supplementary Table S3). GSMM analyses of the gut microbiota suggest that intestinal bacteria can import THCA. However, the identified bacterial GEMs lack reaction or enzyme(s) that can convert αMCA to HCA and HDCA (Supplementary Fig. S5 and Supplementary Dataset 1). Thus, the applied GSMM in the current version of the AGORA compendium failed to unravel gut bacterial pathways related to biosynthesis of C-6 hydroxylated BAs.

### Bioinformatics search for gut bacterial genes involved in biosynthesis of C-6 hydroxylated bile acids

We attempted to identify Clusters of Orthologous Groups (COGs) that correlated to a ratio of two closely related sets of BAs, ignoring conjugation, termed ‘BAGs’, as described in (Methods). Ratios of closely related BAGs were correlated to MGSs by Spearman’s correlation and differences in correlation strength were evaluated by Wilcoxon test. One thousand four hundred and twenty three COGs were associated (FDR corrected *P* < 0.05) with one or more BAG ratios (Supplementary Table S4). When investigating the degree of interconnectedness between COGs that significantly correlated with a given BAG ratio, we failed to identify any interconnected and functionally related subclusters of COGs. Hence, due to a number of shortcomings for annotations in available gene and protein databases, we were unable to identify gut bacterial enzymes that can synthesize human C-6 hydroxylated BAs. When employing a χ^2^-test to investigate the dependency between direction of correlation with a given BAG ratio as evaluated by Spearman’s correlation and the presence or absence of a COG for all MGSs; no COG was found to have significant (FDR corrected *P* < 0.05) dependencies. Additional information on the applied approach is given in (ESM).

## Discussion

There is a major variation in the circulating BA profile among mammalians. The primary BAs in humans are CA and CDCA whereas in rodents and especially in pigs, the dominant BAs are HCA, HDCA, and their conjugates^1,2,15,26–29^. Thus in humans, the C-6-hydroxylated HCA, HDCA and derivatives occur in much smaller amounts in blood than in animals. When it comes to the site of origin, CA and CDCA are in various mammalians including humans synthesized in the liver from cholesterol by two pathways. In the classical pathway, the CYP7A1 is the rate-limiting step while CYP27A1 is rate-limiting in the alternative pathway. In contrast, the site of biosynthesis of HCA and HDCA in humans and animals may be different. There is experimental evidence that HCA can be synthesized from CDCA, and HDCA from TLCA and LCA in human liver microsomes through the CYP3A4-mediated 6α-hydroxylation pathway. Whether the human gut microbiome is involved in the synthesis of HCA and its C-6 hydroxylated derivatives is unsettled. In rats, HDCA can be produced via microbial processing of β-muricholic acid or derived from LCA by liver enzymes that convert LCA to 3α,6 β-dihydroxy cholanoic acid that by bacterial biotransformation is converted to 3α-hydroxy-6-keto cholanoic acid, and finally reduced to HDCA.

Here, we tested the hypothesis that components of the human microbiome contribute to the biosynthesis of C-6-hydroxylated BAs. Intriguingly, we demonstrated significant positive associations between distinct communities of *Clostridia* species and the serum level of C-6 hydroxylated BAs. Obviously, the cross-sectional study design did not allow disentangling the directionality of the relationship. We next applied a metagenomics approach to make probable the existence of gut bacterial enzymes involved in the biotransformation of C-6 hydroxylated BAs but our analyses fell short. Still, the metagenomics analyses do not exclude the possibility for bacterial biosynthesis of C-6-hydroxylated BAs in humans since there is a major lack of functional annotation of bacterial genes in currently available databases. Similarly, GSMM of the human gut microbiota based on available limited literature and database knowledge was unable to identify the microbial reactions or enzymes that can convert αMCA to HCA or HDCA, and their derivatives. Therefore, a further clarification of a putative gut microbiome contribution to the origin of C-6-hydroxylated BAs in human serum must await the development of more complete catalogs of functionally annotated bacterial genes.

Our findings of inverse relationships between C-6-hydroxylated BAs and markers of dysmetabolism and adiposity in 283 nondiabetic Danes extend previous findings in both humans and pigs. Thus, in a US study of 31 morbidly obese individuals who had undergone sleeve gastrectomy, fasting and post lipid meal increase in plasma concentration of glycine- conjugated HCA correlated positively with the surgical weight loss at 12 weeks of follow-up^28^. In a French study of 205 individuals with prediabetes, the plasma total HCA/CDCA ratio (a marker of C-6 hydroxylation activity) correlated inversely with BMI and HOMA-IR^26^. Similarly, in a Chinese study comprising 20 healthy, 18 prediabetic and 17 drug-naïve type 2 diabetes individuals, the authors reported inverse correlations between C-6 hydroxylated BAs and BMI and plasma glucose levels after an oral glucose load^27^. In studies of pigs and mice supplemented with experiments in enteroendocrine L-cells, the same investigators showed that HCA, HDCA and conjugated derivatives add to promote metabolic health via an enhanced GLP-1 biosynthesis and secretion involving an activation of TGR5 and an inhibition of FXR^27^. Hence, given the critical role of C-6-hydroxylated BAs in maintaining metabolic homeostasis, the novel insights call for future testing of potential therapeutic applications of these BAs.

Our study has limitations. The study design is observational and no causality can be inferred. Moreover, the present study is like epidemiological studies of gut microbiota in general limited by not being able to sample microbiota specimens at the optimal intestinal localization. The gut microbiota in the small intestine has the first exposure to BAs, while our microbiota analyses were performed on faecal samples as a proxy for the overall intestinal microbiota. Additionally, serum BAs are subject to first-pass liver metabolism, which is likely to affect circulating BAs to various extents, adding another element of variability^3^.

## Conclusion

Co-abundant species of *Clostridia* in the human gut microbiota are positively associated with fasting serum of C-6 hydroxylated BAs, which in turn are inversely associated with adiposity traits and markers of dysmetabolism. In perspective, our findings merit testing in future preclinical interventions and human feasibility studies exploring the potential therapeutic role of C-6 hydroxylated BAs in states of obesity and its metabolic co-morbidities. In addition, when human gut metagenome databases become considerably more complete, further search for a bacterial contribution to the biosynthesis of C-6-hydroxylated BAs merits consideration.

## Supplementary Information

Supplementary Information 1.

Supplementary Information 2.

Supplementary Information 3.

## Data Availability

Raw shotgun metagenomics data that support the findings of this study have been deposited in the European Nucleotide Archive (accession numbers: ERP003612, ERP004605) with public access. Correspondence and requests for data used in this study are directed to Prof. Oluf Pedersen.
