# Supplementary material for: Conjugated C-6 hydroxylated bile acids in serum relate to human metabolic health and gut Clostridia species
Source: Sci Rep. 2021 Jun 24;11:13252. doi: 10.1038/s41598-021-91482-y (PMC8225906; doi:10.1038/s41598-021-91482-y)
Supplement: Supplementary file 1 — Supplementary Information 1. [file 41598_2021_91482_MOESM1_ESM.docx]

**Electronic Supplementary Materials (ESM)**

**Conjugated C-6 hydroxylated secondary bile acids in serum relate to human metabolic health and gut *Clostridia* species**

Anders Ø Petersen ^a,1^, Hanna Julienne ^b, 1^, Tuulia Hyötyläinen ^c^, Partho Sen ^d^, Yong Fan ^e^, Helle K Pedersen ^e, f^, Sirkku Jäntti ^g^, Tue H Hansen ^e^, Trine Nielsen ^e^, Torben Jørgensen ^h^, Torben Hansen ^e^, Pernille Neve Myers^a^, H. Bjørn Nielsen ^f^, S. Dusko Ehrlich ^b^, Matej Orešič ^d, i^, and Oluf Pedersen ^e, *^

**Affiliations**

^a^ Department of Health Technology, Technical University of Denmark, 2800 Lyngby, Denmark

^b^ Metagenopolis, INRA, AgroParisTech, Université Paris-Saclay, and Institut Pasteur, Bâtiment Yersin 5ème étage 28 rue du Docteur Roux, 75015 Paris, France

^c^ School of Science and Technology, Örebro University, Örebro, Sweden

^d^ Turku Bioscience Centre, University of Turku and Åbo Akademi University, FI-20520 Turku, Finland

^e^ Novo Nordisk Foundation Center for Basic Metabolic Research, Faculty of Health and Medical Sciences, University of Copenhagen, Blegdamsvej 3B, 2200 Copenhagen, Denmark

^f^ Clinical Microbiomics A/S, Ole Maaløes Vej 3, 2200 Copenhagen, Denmark

^g^ Drug Research Program, Division of Pharmaceutical Chemistry and Technology, Faculty of Pharmacy, University of Helsinki, 00014 Helsinki, Finland

^h^ Center for Clinical Research and Prevention, Frederiksberg Hospital, Copenhagen, Denmark

^i^ School of Medical Sciences, Örebro University, 702 81 Örebro, Sweden

^1^ The two authors contributed equally

**^*^ Corresponding author**

Oluf Pedersen, Novo Nordisk Foundation Center for Basic Metabolic Research, Faculty of Health and Medical Sciences, University of Copenhagen, Blegdamsvej 3B, 2200 Copenhagen, Denmark Email: [oluf@sund.ku.dk](mailto:oluf@sund.ku.dk)

### Genome-Scale Metabolic Modeling (GSMM)

### Background

Previously, genome-scale metabolic models (GEMs) combined with the AGORA tool has been used to estimate metabolic capabilities of the gut microbiota in carbohydrate and short-chain fatty acid metabolism. Recently, these GEMs were expanded to include bile acid (BA) metabolic pathways, and thereby linking the gut bacteria with the BA metabolism in human health and disease^1, 2^.

To reduce the complexity of GSMM of BA metabolism facilitated by human gut microbiota, we selected highly abundant species from each bacterial community, *i.e.* MGS. GEMs of 26 abundant bacterial species (MGSs with > 500 bacterial genes), were retrieved from the AGORA_BA compendium (v1.02), stored at the Virtual Metabolic Human Database (VMH)^2, 3^ (<https://vmh.uni.lu/)>, and assessed for further analysis (**Supplementary Figure 6**). Three types of GSMM strategies were adopted, (i) Modeling the primary growth of individual microbe based on Average European Diet (AED)^2^, (ii) Microbe-microbe pairing (co-growth) to determine the joint ability of the microbes for BA biotransformation, and (iii) Personalized community modeling at an individual (obese or non-obese) level using metagenomics data. GSMM was performed using COBRA Toolbox^4^ deployed in MATLAB *Inc.,* version R2017a.

#### Analyses of intestinal microbial communities in 283 Danish adults applying the genome-scale metabolic modeling tool combined with the AGORA compendium

All possible pairs among bacterial species were established using the ‘joinModelsPairwiseFromList()’ function implemented in the Microbiome Modeling Toolbox (MMT)^5^. An ‘unknown’ model that contains common reactions and metabolites of the abundant species (MGSs with > 500 genes) other than the 26 identified bacteria was added. The model maintains the repertoire of metabolic reactions in the gut ecosystem, which is otherwise under-determined. A common luminal compartment ‘[u]’ was introduced that enabled metabolite exchanges and cross-feeding between the bacteria (**Supplementary Figure 8**). The composition of the Average European Diet (AED) was retrieved from Virtual Metabolic Human (VMH; https://www.vmh.life/). The intestinal luminal uptake rates were constrained by the dietary fluxes using ‘useDiet()’. The paired models were allowed to exchange conjugated bile acids (BAs) while the uptake of other metabolites was limited. Different types of metabolic interactions were estimated by ‘simulatePairwiseInteractions()’ deployed in MMT with a minimum growth rate difference of 10% between the microbes. Each microbial model (GEM) was allowed to grow on its own and as a pair under anaerobic conditions. Flux Variability Analysis (FVA) using ‘fastFVA()’^6^ was performed to assess maximum and minimum fluxes incurred by BA reactions in the paired model and the flux span was estimated. The paired models that carried fluxes through the BA exchange reactions were selected and grouped by their genus.

Several metabolic reconstruction/models such as Recon3D^7^, the small intestinal epithelial cells– (sIECs)^8^ model, and the VMH database and bibliographic references^9-11^, were mined and putative BA transporters in the human gut were identified. A metabolic model of human luminal/intestinal cells (*hLC_001*) was reconstructed from Recon3D as a reference model. The BA transporters and exchange reactions were added. Sanity checks were performed using COBRA Toolbox^4^. All models carried out basic metabolic tasks, including exchange and transport of BAs. The lists of reactions and metabolites in *hLC_001* are provided in (**Supplementary Dataset 1**). The host model (*hLC_001*) was coupled with microbial models using ‘createMultipleSpeciesModel()’ coded in MMT-COBRA toolbox^4, 5^ and necessary coupling constraints were added. Similarly, a common lumen compartment ‘[u]’ was added for the metabolite exchanges between bacteria and luminal epithelial cells. A compartment ‘[b]’ for body fluids was introduced. For each of the 283 study individuals a community/consensus model of the 26 chosen bacteria was developed as outlined by Baldini *et al.,*^5^. The average reactions and metabolites of the consensus model was 15,800 and 13,900 respectively.

The individual contribution of a bacteria in a community to BA production was estimated by the following steps, i) the faecal production rate was maximized, ii) flux variability analysis (FVA) was performed on the BA exchange reactions of each species and flux span was calculated, and iii) the estimated metabolic potential (EMP) was computed as given in (eq.1). The EMP of a BA reaction is given by EMP_ij_ = A_i_ × v_j_ (eq.1), where 'EMP_ij_' denotes the estimated metabolic potential of 'j^th^' BA in 'i^th^' species. A and v represent the relative abundance of a species and absolute flux potential (mmol/gDw/hr), respectively^12^. EMP is a measure to quantify the metabolic efficiency of a particular reaction, under a specified condition^12^. The total EMP determines the metabolic capability/potential of the bacteria to perform a particular function. In this study, EMP was used to evaluate the performances of bacteria for BA transformation. For any particular BA, EMP was used to estimate the flux potential, the uptake or compound produced by bacteria. Overall, the maximum luminal uptake (LUP) and secretion (LSP) potential of the BA exchange reactions, facilitated by the gut bacteria in the community model, was estimated and adjusted with the abundances of MGSs. BA reaction abundances were estimated by ‘calculateReactionAbundance()’.

#### Metabolic modeling of human gut microbiota for bile acid transformation

In non-obese individuals, the serum levels of CA and CDCA were positively correlated (ρ=0.21, p < 0.05) with their luminal secretion potential (LSP) (**Supplementary Figure 9A**), whereas in the obese individuals, these associations were weak or absent. However, conjugates of CAs such as GCA, TCA correlated directly with their LSP (**Supplementary Figure 9B**). In addition, in non-obese individuals, the serum CA concentration correlated directly with the estimated metabolic potentials (EMPs) (see **Methods**) of *Ruminococcus spp.* and *Bacteroides spp.*, respectively, for the TCA exchanges. Likewise, serum CDCA correlated positively with the EMPs of the *Eubacterium spp.*, *Parabacteroides spp.*, and *Bifidobacterium spp.*, respectively, for the GCDCA exchange (**Supplementary Figure 10)**. In contrast, in the obese group the serum CA and CDCA levels correlated positively with the EMPs of *Ruminococcus_lactaris_ATCC_29176* and *Bacteroides_massiliensis_B846dnLKV334*, respectively (**Supplementary Figure 11)**.

As a common denominator, the abundances of *Ruminococcus* *spp.*, and *Bacteroides spp.,* may determine the BA biotransformation in non-obese and obese individuals (**Supplementary Figure 10** and **Supplementary Figure 11)**. The total EMP of the luminal BA exchange reactions carried by *Bacteroides_coprocola_M16_DSM_17136* was increased (p = 0.040), whereas that of *Ruminococcus_bromii_L2_63* was decreased (p = 0.008) in the obese group (**Supplementary Fig. 12**). Additionally, in the obese subjects, the EMP for TCA exchange by *Coprococcus_eutactus_ATCC_27759* correlated positively with most of the serum BAs (except CA and CDCA) measured in this study (**Supplementary Fig. 11**). Simulation of the pairwise interaction between these bacteria suggests that, most of the studied taxa exhibit Amensalism (**Supplementary Fig. 13B**).

The serum DCA concentration correlated directly with their LSPs in non-obese and obese individuals (**Supplementary Fig. 9**). Interestingly, the serum level of 7-oxo-DCA (a stable intermediate) correlated with the LSPs of primary and secondary BAs in non-obese people (**Supplementary Fig. 9A**). However, a similar association was not observed in the obese subjects (**Supplementary Fig. 9B**)**.** The conjugates and intermediates of serum DCAs correlated positively with EMP of *Ruminococcus spp.,* for the exchange of GCA in the non-obese group (**Supplementary Fig. 9A**), while they correlated positively with the EMPs of *Coprococcus eutactus* for the exchange of TCA in the obese subjects (**Supplementary Fig. 9B**). No significant correlation was observed between serum LCA and their LSP in neither non-obese nor obese individuals (**Supplementary Fig. 9**).

UDCA comprises approximately 4% of the serum BA pool in humans^13^. In non-obese subjects, LSP of UDCA correlated directly with the serum levels of its precursor CDCA (**Supplementary Fig. 9A**) and inversely with its serum conjugates. Findings, which are suggesting that UDCA may have been absorbed by the epithelial cells or eliminated in the stool. Similar associations were not observed in obese. Serum UDCA levels were directly correlated with its precursor CDCA exchanged by *Eubacterium_siraeum_70_3* or Bacteroides*_xylanisolvens_XB1A* in non-obese and obese people, respectively (**Supplementary Fig. 10** and **Supplementary Fig. 11**).

No significant correlation was observed between serum LCA or GLCA and their LSPs in the non-obese and obese subjects (**Supplementary Fig. 9**). LCA can be produced from CDCA and UDCA by 7α/7β-dehydroxylation pathway or alternatively de-conjugated from GLCA. In obese subjects, serum GLCA and LCA concentrations correlated directly with the GCDCA uptake potential of *Ruminococcus_bromii_L2_63*, *Lachnospiraceae_bacterium_sp_5_1_63FAA*,

*Bacteroides_plebeius_M12_DSM_17135* and *Roseburia_hominis_A2_183*, respectively (**Supplementary Fig. 11**). In non-obese individuals, LCA was correlated with the TCDCA uptake potential of *Ruminococcus_sp_5_1_39BFAA*, *Bifidobacterium_adolescentis_ATCC_15703* and *Ruminococcus_sp_SR1_5* (**Supplementary Fig. 10**). UDCA can be synthesized from LCA by 7α dehydroxylation pathway, CDCA by hydroxysteroid dehydrogenase (7α/7 β HDSH) or from deconjugation of TUDCA and GUDCA (**Supplementary Fig. 5).** UDCA was negatively and positively correlated with TCDCA uptake potential of *Ruminococcus_sp_5_1_39BFAA* and *Bacteroides_plebeius_M12_DSM_17135*, respectively (**Supplementary Fig. 11**). UDCA and its conjugates correlated directly with the CDCA secretion potential of *Bacteroides_xylanisolvens_XB1A* and with the GCDCA uptake potential of *Coprococcus_eutactus_ATCC_27759* (**Supplementary Fig. 11**).

THCA and GHCA can be de-conjugated or hydrolyzed to HCA. In rodents, TαMCA can be deconjugated to αMCA, which can produce HCA and HDCA^14^. We have not identified microbial enzymes that could facilitate these conversions. However, we putatively identified gut transporters for THCA (**Supplementary Dataset 1**)^6, 8, 9, 15, 16^. THCA and GHCA in serum of non-obese and obese subjects correlated directly with their luminal uptake potential and with serum TαMCA concentration while HCA correlated directly with unconjugated primary and secondary BAs in the lumen (**Supplementary Fig. 9A** and **B**). In addition, the GCDCA uptake potential of *Parabacteroides_sp_D13* correlated positively with the TαMCA serum level in non-obese subjects (**Supplementary Fig. 10**). Based on these findings, we cannot exclude that metabolic biotransformation of MCA might take place the human gut.

Furthermore, we calculated the possible non-redundant pairwise interactions among 26 bacterial species subjected to Average European Diet (AED)^2, 3^. These pairs were constrained for uptake and secretion of unconjugated and conjugated BAs, respectively. Most of the species pairs that have the ability to transform or deconjugate BAs showed Amensalism i.e, in the paired simulation, one microbe grows slower than the other but their growth is not mutually dependent^2^ (**Supplementary Fig. 13B**). Twenty-one species pairs that can transform BA belonged to the *Ruminococcus* and *Bacteroides* genera **(Supplementary Fig. 13A**). These bacteria might be potential markers for biotransformation of some BAs.

### Construction of communities of human intestinal species

Metagenomics yields high dimensionality datasets where the number of variables by far exceeds the number of samples. This situation is prone to major pitfalls, *i.e.* lack of model generalization and difficulties in data interpretation. We therefore chose to apply a form of feature agglomeration to reduce the dimensionality of our microbiome dataset, *i.e.* we grouped communities of species based on their co-abundance. A similar co-abundance grouping has previously been applied with success at the genus level^17^. To form groups of co-abundant species we applied a pipeline of co-abundance graph inference (see **Methods**) and used community detection on the network to group the species. The modularity of the community partition of the largest connected component reached a value of 0.69 (theoretical maximum is 1), indicating an acceptable match between the community partition and the graph structure.

While yielding a view coherent with the enterotype approach, the community approach is conceptually very different. *Communities are clusters of species, whereas enterotyping clusters individuals.* Therefore, an individual can only belong to one enterotype, whereas an individual can have a moderate to high level of several communities. The community approach overcomes several issues related to enterotypes^17, 18^, being continuous, robustly detected, and usable as features in machine learning algorithms. In the current context, the community C is particularly interesting because it groups together species known to be health promoting such as *Akkermansia municiphila^19^* and *Faecalibacterium^20^* and it correlates with serum concentration of secondary BAs.

### Search for enzymes encoded by the human gut microbiome and involved in biosynthesis of C-6 hydroxylated bile acids

In an attempt to identify bacterial genes with the capability of producing C6-hydroxylated BAs, different types of analyses were undertaken.

First, we undertook a correlation-based analysis attempting to identify Clusters of Orthologous Groups (COGs) of proteins that might be responsible for the conversion of one hydroxylation pattern into another. The assumption was that existence of gut bacteria that are able to shift the concentration ratio of substrate:product would allow identification of bacteria that correlate strongly to a certain BA ratio. We summarized BAs into BA groups (BAGs), by pooling a given hydroxylation pattern regardless of conjugation (e.g. TCA, GCA and CA into “CAs”) and using their sum concentration as the concentration for that BAG. Pairs of BAGs that differed only in one hydroxyl group were identified as likely substrate:product pairs and the ratio of their concentrations were computed for each individual. These BAG ratios were linked to the abundance of MGSs by Spearman correlation analysis. In these analyses we assumed that muricholic acid (MCA) and hyocholic acid (HCA) were produced from chenodeoxycholic acid (CDCA). However, following Benjamini-Hochberg (FDR) correction, no MGS was significantly (p<0.05) correlated to ratios between HCAs and CDCAs or MCAs or CDCAs, respectively.

It is of note that well-studied BA metabolism pathways did in fact in these analyses show significant results. Both the ratio of CA to deoxycholic acid (DCA) and CDCA to ursodeoxycholic acid (UDCA) returned significantly (FDR corrected p<0.05) correlated MGSs. In an attempt to identify COGs that correlated with the variables of interest, Spearman correlation strengths of MGSs containing a given COG were compared with those where the COG was absent by Wilcoxon testing. We annotated bacterial genes to COGs using EggNOG 4.5. After FDR correction, several COGs correlated significantly (q<0.05) to both well-studied and hypothetical BA ratios. The number of significantly (q<0.05) correlating COGs is reported in (**Supplementary Table 4**). Manual inspection of COG annotations showed that the plurality of the COGs was associated with cell membrane integrity, antibiotic resistance and other functions that relate to the antibacterial nature of BAs.

Additionally, a list of known BA-related genes (supplementary file all_fastas.fa) was mapped using eggNOG (v4.5.1) and the COGs that correlated to BAGs were inspected for the presence of known BA-related genes. However, none of these genes were significantly associated with any BAG ratio of interest. In a final attempt to identify narrow the search space of significantly related COGs, the *STRING* database was employed, which evaluates the relatedness between two COGs by a score between 0 and 1, 1 being highly correlated, 0 meaning no relation. The COGs that significantly correlated to a given BAG ratio were identified in the *STRING* database, and relatedness was evaluated as the sum of intermediary weights. Any connection with spanned more than one intermediary COG was considered tenuous and ignored. No BAG ratio was correlated to a set of COGs that showed noticeable degrees of interconnectedness in the STRING database. This analysis was also carried out with a lower significance threshold (FDR corrected p<0.01) but with similar results.

Lastly, Dr. Solenne Marion of École Polytechnique Fédérale de Lausanne, Switzerland kindly provided us with gut bacterial strains that when given to germfree mice produced secondary murine bile acids such as β MCA. In an attempt to identify novel enzymes involved in muricholic BA biosynthesis, we made predicting studies using GeneMark on these specific bacterial genomes. The genes were blasted against a list of known BA-related genes (supplementary file all_fastas.fa) and the score (1/e-value) was plotted against the gene position. However, we failed to identify any bacterial genes encoding novel enzymes involved in biosynthesis of muricholic acids.

### Logistic ridge regression

Each of the training and test sets were stratified to have the same proportion of obese individuals included in it, which was used to calculate the area under the receiver operating characteristic curve (AUC). We computed the model’s coefficients on the training set and the model performance on the test set for each partition. We then used the 10,000 model coefficients to compute a mean coefficient and the standard deviation for each BA of the coefficient. The 10,000 models were used to compute a mean AUC estimate with its variability. Regularized regression techniques were employed, because they perform well in cases when the dependent variable is noisy and the number of samples is modest as compared with the number of examined variables^21^.

###

### Construction of heatmaps and correlation testing

Spearman correlations were computed with R and the associated p-value with ‘*cor.test’*. Multiple testing correction was applied with the ‘*fdrtool’* package

(<http://strimmerlab.org/software/fdrtool/>). A community was associated with for instance an adiposity trait if the community correlated (after multiple testing correction) with at least one negative association with one of the following traits: Homeostatic Model Assessment of Insulin Resistance (HOMA-IR), BMI, waist circumference, waist to hip ratio or body fat percentage.

### Graph based community construction

Following MGS construction, following steps were taken:

1. Selection of MGS that were present in at least 5% of samples and had a mean abundance value of fragments per kilobase per million mapped reads (FPKM) of 10 to 7 across samples.
2. For the selected MGSs, the co-abundance graph was inferred with the SPIEC EASI pipeline.
3. We constructed the community structure with the walktrap algorithm from the igraph R package (<http://igraph.org/r/>).
4. The graph structure was transferred to a Gephi https://gephi.org/ compatible format.
5. The community abundances were computed by summing the abundance of MGSs in the community.

The computational processes were launched applying the Gleesso pipeline function with the following set of parameters: occurence_treshold of 0.05; abundance_treshold of 10^−7^; and lambda.min.ratio of 0.01.

### Laboratory notes

**Serum sample preparation**

Internal standards (4 µl of internal standard mixture: 500 ppb for LCA-d4 and 50 ppb for others) were added to 20 µl of serum, and 24 µl of acetonitrile was added to precipitate proteins. The samples were first vortexed, then put to the ultrasound for three minutes, and finally centrifuged (5 min, at 12100 G). The sample was evaporated to dryness, and reconstituted to 4 µl of methanol, and diluted with 6 µl of water. Solution was analyzed as such, and as dilution of 1 to 20 with the same solvent.

### Ultra-performance liquid chromatography-tandem mass-spectrometry (UPLC-QqQMS) of bile acids in serum samples

Analysis was performed on an Acquity UHPLC system, Waters (Milford, MA, USA) and Waters Xevo TQ-S MS (Manchester, UK). Chromatography was performed using an Acquity HSS T3 (2.1 x 100 mm, 1.7 μm) column, Waters (Milford, USA), kept at 35 ^o^C. Injection volume was 5 µL. Separation was performed using gradient elution with 0.1 % formic acid in water (v/v) (A) and 0.1% formic acid in acetonitrile: methanol (3:1, v/v) (B) at a flow rate of 0.5 mL/min. Gradient program was 0 min 15 % B, 1 min 30 % B, 16 min 70 % B, 18-20 min 100 % B. Mass spectrometry was performed in negative polarity using the capillary voltage of 2.0 kV. Desolvation temperature was 650 ^o^C, and the source temperature was 150 ^o^C. Analytes were detected using selected reaction monitors (SRM) using auto dwell time function (dwell time of 20-95 ms, 20 points/peak). Analytes were quantified by an internal standard method. Control samples were run as every seventh sample monitoring the quality of analyses.

### Reagents and standards used in analyses of serum bile acids

Abbreviations of analytes are presented in (**Fig. 2A**). CA, CDCA, DCA, DHCA, GCA, GCDCA, LCA, TCA, TCDCA, TDCA, TDHCA, THCA, THDCA, TLCA, and TUDCA were obtained from Sigma-Aldrich (St. Luis, Missouri, U.S.A), HDCA, HCA, αMCA, β MCA, ωMCA, 7-oxo-HDCA, 7-oxo-DCA, 12-oxo-LCA, TαMCA, T β MCA, TωMCA, GDHCA, GHCA, and GHDCA from Steraloids (Newport, RI, U.S.A), GLCA and GUDCA from Calbiochem (Gibbstown, NJ, U.S.A), and GDCA and UDGA from Fluka (Buchs, Switzerland). Internal standards, CA-d4, LCA-d4, UDCA-d4, CDCA-d4, DCA-d4, GCA-d4, GLCA-d4, GUDCA-d4 and GCDCA-d4 were obtained from Qmx laboratories (Essex, UK) except TCA-d4, which was synthesized in our laboratory.

### Detection of unknown serum bile acids

MS conditions used for the detection of 28 unknown BAs are presented in ^22^ and in (**Supplementary Table 5**). Shortly, 15 of these unknown BAs were detected by using SRM transitions targeting MCAs, HCA, CA, or their conjugates, 11 using the SRM transitions of UDCA, HDCA, DCA, CDCA, or their conjugates, and two using the SRM transitions of oxo-BAs, respectively. UHPLC-MS/MS spectra and accurate mass with UHPLC-QTOFMS were determined for all the unknowns. Accurate mass determined by UHPLC-QTOFMS corresponded also with the structures of taurine BA, glycine BA, and intact BA. Taurine conjugates showed typical fragmentation pattern with ions at *m/z* 80, 107, and 124, and loss of water from parent structure. Glycine conjugates showed glycine fragment at m/z 74, and sequential losses of H2O, CO2, H 2CO2, or H2 from parent BA structure^22^. Intact BAs had similar sequential losses.

### Supplementary tables

## Supplementary Table 1

|  | **Men** | **Women** | **p-value** |
| --- | --- | --- | --- |
|  | N=133 | N=150 |  |
| Waist to hip ratio (-) | 1.0 (0.1) | 0.9 (0.1) | <0.001 |
| BMI (kg/m²) | 29.5 (5.5) | 29.6 (6.4) | 0.904 |
| Body fat (%) | 26.3 (6.4) | 34.8 (7.0) | <0.001 |
| S-Leptin (µg/l) | 8.4 (9.7) | 31.4 (33.4) | <0.001 |
| S-Adiponectin (mg/l) | 8.6 (5.2) | 13.4 (8.0) | <0.001 |
| P-Cholesterol (mmol/l) | 5.4 (1.0) | 5.5 (0.9) | 0.462 |
| P-HDL cholesterol (mmol/l) | 1.4 (0.5) | 1.6 (0.5) | 0.001 |
| P-LDL cholesterol (mmol/l) | 3.4 (0.9) | 3.3 (0.8) | 0.272 |
| P-Triglyceride (mmol/l) | 1.3 (0.7) | 1.3 (0.7) | 0.942 |
| P-Glucose (mmol/l) | 5.8 (0.5) | 5.7 (0.6) | 0.078 |
| C-peptide (pmol/l) | 809.0 (366.0) | 786.0 (394.0) | 0.616 |
| S-Insulin (pmol/l) | 53.7 (34.9) | 52.5 (34.7) | 0.781 |
| HOMA-IR adjusted for BMI | -0.1 (1.0) | 0.1 (1.3) | 0.207 |
| HOMA-IR (mU/l * mM) | 2.0 (1.4) | 2.0 (1.4) | 0.692 |
| P-C-reactive protein (mg/l) | 2.0 (1.9) | 3.1 (3.2) | <0.001 |
| S-Interleukin 1 receptor antagonist (ng/l) | 418.0 (366.0) | 535.0 (454.0) | 0.017 |
| S-Interleukin 6 (ng/l) | 17.8 (21.3) | 21.3 (21.4) | 0.169 |
| S-Tumor necrosis factor alfa (ng/l) | 21.9 (32.2) | 28.6 (56.4) | 0.216 |
| B-Lymphocytes (count/l * 10E9) | 1.8 (0.5) | 1.9 (0.5) | 0.225 |
| B-Leucocytes (count/l * 10E9) | 6.2 (1.7) | 6.0 (1.6) | 0.245 |
| S-TCA (ng/mL) | 12.9 (16.4) | 14.4 (38.2) | 0.654 |
| S-TaMCA (ng/mL) | 3.6 (3.5) | 4.2 (5.1) | 0.271 |
| S-THCA (ng/mL) | 1.3 (1.3) | 1.1 (0.9) | 0.148 |
| S-TCDCA (ng/mL) | 24.5 (28.3) | 24.2 (46.8) | 0.935 |
| S-TDCA (ng/mL) | 22.8 (37.8) | 20.4 (31.0) | 0.572 |
| S-TUDCA (ng/mL) | 3.1 (3.0) | 2.7 (2.7) | 0.218 |
| S-GCA (ng/mL) | 77.8 (85.7) | 68.6 (136.0) | 0.491 |
| S-GHCA (ng/mL) | 5.6 (6.3) | 4.1 (3.9) | 0.018 |
| S-GUDCA (ng/mL) | 21.0 (23.9) | 20.1 (39.3) | 0.817 |
| S-GCDCA (ng/mL) | 179.0 (153.0) | 156.0 (165.0) | 0.233 |
| S-GDCA (ng/mL) | 86.2 (114.0) | 68.2 (82.3) | 0.131 |
| S-GHDCA (ng/mL) | 1.7 (1.5) | 1.5 (1.0) | 0.215 |
| S-GLCA (ng/mL) | 4.5 (5.6) | 4.8 (6.5) | 0.693 |
| S-CA (ng/mL) | 92.5 (176.0) | 91.1 (176.0) | 0.944 |
| S-HCA (ng/mL) | 5.6 (4.4) | 4.5 (4.4) | 0.046 |
| S-7oxo-DCA (ng/mL) | 2.7 (7.4) | 2.0 (4.8) | 0.368 |
| S-UDCA (ng/mL) | 11.2 (12.8) | 13.8 (28.8) | 0.311 |
| S-CDCA (ng/mL) | 66.5 (113.0) | 85.7 (165.0) | 0.251 |
| S-DCA (ng/mL) | 108.0 (87.2) | 105.0 (96.4) | 0.765 |
| S-LCA (ng/mL) | 7.4 (4.4) | 7.1 (6.3) | 0.667 |
|  |  |  |  |

**Supplementary Table 1: Summary statistics stratified by sex of bio-clinical variables including fasting serum concentrations of bile acids in 283 individuals**. Values indicate mean and standard deviation (SD) for metabolic and inflammatory related clinical traits; p-value is obtained by performing a t-test. BMI is body mass index. Prefix P- and S- indicate plasma and serum, respectively. BA abbreviations and complete compound name are: HCA - Hyocholic acid, HDCA - Hyodeoxycholic acid, αMCA - α-muricholic acid, 7oxo-DCA - 7-oxo-deoxycholic acid, DCA - Deoxycholic acid, CA - Cholic acid, LCA - Lithocholic acid, CDCA - Chenodeoxycholic acid, UDCA - Ursodeoxycholic acid). Abbreviated names preceded with G or T indicate that the BA is conjugated with a glycine or a taurine, respectively.

## Supplementary Table 2

|  |  | **BMI** | **Leptin** | **HOMA-IR** |
| --- | --- | --- | --- | --- |
| **Men** | **AUC** | 0.71±0.1 | 0.47±0.12 | 0.69±0.1 |
|  | **p-value** | 0.017 | 0.58 | 0.03 |
| **Women** | **AUC** | 0.70±0.09 | 0.63±0.1 | 0.70±0.9 |
|  | **p-value** | 0.024 | 0.09 | 0.017 |

**Supplementary Table 2:**  **Outcome of Logistic Ridge Regression (LR) modeling of bile acids-driven classification by the targets of body mass index (BMI), fasting serum leptin concentration and the insulin resistance measure, HOMA-IR, in 150 women and 133 men.**Mean area under the curve (AUC) and AUC standard deviation of Ridge classification models on the 10,000 different validation sets. The empirical p-value is reported if the AUC was higher than 0.50 (the empirical p-value consists of the fraction of subsets with an AUC inferior to 0.50).

## Supplementary Table 3

|  | **Non-obese (BMI < 27)** | **Obese (BMI > 27)** | **p-value** |
| --- | --- | --- | --- |
|  | N=106 | N=177 |  |
| Waist to hip ratio (-) | 0.8 (0.1) | 0.9 (0.1) | <0.001 |
| BMI (kg/m²) | 22.9 (1.8) | 33.5 (3.5) | <0.001 |
| Body Fat (%) | 24.0 (5.9) | 34.8 (6.1) | <0.001 |
| S-Leptin (µg/l) | 6.7 (8.5) | 28.9 (31.6) | <0.001 |
| S-Adiponectin (mg/l) | 14.0 (7.7) | 9.4 (6.4) | <0.001 |
| P-Cholesterol (mmol/l) | 5.5 (1.0) | 5.5 (1.0) | 0.956 |
| P-HDL cholesterol (mmol/l) | 1.9 (0.6) | 1.4 (0.4) | <0.001 |
| P-LDL cholesterol (mmol/l) | 3.2 (0.8) | 3.4 (0.8) | 0.012 |
| P-Triglyceride (mmol/l) | 1.0 (0.4) | 1.5 (0.8) | <0.001 |
| P-Glucose (mmol/l) | 5.5 (0.5) | 5.9 (0.5) | <0.001 |
| C-peptide (pmol/l) | 508.0 (177.0) | 971.0 (364.0) | <0.001 |
| S-Insulin (pmol/l) | 28.4 (13.6) | 68.0 (35.1) | <0.001 |
| HOMA-IR adjusted for BMI | -0.1 (0.8) | 0.03 (1.4) | 0.348 |
| HOMA-IR (mU/l * mM) | 1.0 (0.5) | 2.6 (1.5) | <0.001 |
| P-C-reactive protein (mg/l) | 1.2 (1.1) | 3.4 (3.0) | <0.001 |
| S-Interleukin 1 receptor antagonist (ng/l) | 348.0 (382.0) | 559.0 (420.0) | <0.001 |
| S-Interleukin 6 (ng/l) | 15.7 (18.6) | 22.1 (22.6) | 0.010 |
| S-Tumor necrosis factor alfa (ng/l) | 20.5 (37.0) | 28.3 (51.4) | 0.145 |
| B-Lymphocytes (count/l * 10E9) | 1.8 (0.5) | 1.9 (0.5) | 0.012 |
| B-Leucocytes (count/l * 10E9) | 5.7 (1.8) | 6.3 (1.5) | 0.001 |
| S-TCA (ng/mL) | 12.5 (17.4) | 14.4 (35.4) | 0.541 |
| S-TaMCA (ng/mL) | 4.8 (4.9) | 3.4 (4.1) | 0.014 |
| S-THCA (ng/mL) | 1.6 (1.3) | 1.0 (0.8) | <0.001 |
| S-TCDCA (ng/mL) | 23.9 (26.9) | 24.6 (44.9) | 0.867 |
| S-TDCA (ng/mL) | 22.8 (41.1) | 20.8 (29.6) | 0.651 |
| S-TUDCA (ng/mL) | 2.7 (2.8) | 3.0 (2.9) | 0.347 |
| S-GCA (ng/mL) | 66.5 (64.5) | 76.7 (136.0) | 0.397 |
| S-GHCA (ng/mL) | 6.3 (6.2) | 3.9 (4.3) | 0.001 |
| S-GUDCA (ng/mL) | 17.7 (21.1) | 22.2 (38.2) | 0.212 |
| S-GCDCA (ng/mL) | 165.0 (130.0) | 167.0 (175.0) | 0.904 |
| S-GDCA (ng/mL) | 78.9 (117.0) | 75.4 (86.2) | 0.788 |
| S-GHDCA (ng/mL) | 1.6 (0.9) | 1.6 (1.5) | 0.801 |
| S-GLCA (ng/mL) | 4.8 (7.2) | 4.6 (5.3) | 0.811 |
| S-CA (ng/mL) | 83.4 (163.0) | 96.8 (182.0) | 0.524 |
| S-HCA (ng/mL) | 5.1 (4.2) | 5.0 (4.6) | 0.947 |
| S-7oxo-DCA (ng/mL) | 1.5 (4.4) | 2.8 (7.0) | 0.060 |
| S-UDCA (ng/mL) | 8.5 (11.3) | 15.0 (27.2) | 0.006 |
| S-CDCA (ng/mL) | 63.5 (124.0) | 84.6 (153.0) | 0.208 |
| S-DCA (ng/mL) | 87.0 (80.1) | 118.0 (96.8) | 0.004 |
| S-LCA (ng/mL) | 7.7 (5.2) | 6.9 (5.7) | 0.250 |

**Supplementary Table 3: Summary statistics stratified by body mass index (BMI) of bio-clinical variables including serum bile acids in 106 non-obese and 177 obese individuals.** Values indicate mean and standard deviation (SD) for metabolic and inflammatory related clinical traits; p-value is obtained by performing a t-test. Prefix P and S indicate plasma and serum, respectively. BA abbreviations and complete compound names are : HCA - Hyocholic acid, HDCA - Hyodeoxycholic acid, αMCA - α-muricholic acid, 7oxo-DCA - 7-oxo-deoxycholic acid, DCA - Deoxycholic acid, CA - Cholic acid, LCA - Lithocholic acid, CDCA - Chenodeoxycholic acid, UDCA - Ursodeoxycholic acid. Abbreviated names preceded with G or T indicate that the BA is conjugated with a glycine or a taurine, respectively.

## Supplementary Table 4

| **Category** | **Bile acids group ratio** | **Number of significant (p<0.05) clusters of orthologous groups** |
| --- | --- | --- |
| **Known** | CAs / DCAs | 88 |
|  | CAs / CDCAs | 62 |
|  | CAs / 7-oxo-DCAs | 0 |
|  | DCAs / LCAs | 122 |
|  | 7oxos / DCAs | 64 |
|  | CDCAs / UDCAs | 66 |
|  | CDCAs / LCAs | 0 |
|  | UDCAs / LCAs | 21 |
| **Hypothetical** | CDCAs / HCAs | 73 |
|  | CDCAs / aMCAs | 4 |
|  | aMCAs / HCAs | 2 |
|  | HDCAs / HCAs | 4 |
|  | HDCAs / LCAs | 0 |
| **Unlikely** | CAs / UDCAs | 79 |
|  | CAs / LCAs | 2 |
|  | DCAs / CDCAs | 58 |
|  | 7-oxo-DCAs / CDCAs | 34 |
|  | 7-oxo-DCAs / UDCAs | 71 |
|  | 7-oxo-DCAs / LCAs | 1 |

**Supplementary Table 4: Numbers of clusters of orthologous groups (COGs) of proteins predicted from the gut microbiome of 283 individuals showing significant correlations with bile acids ratio groups.** BA abbreviations and complete compound name are: HCA - Hyocholic acid, HDCA - Hyodeoxycholic acid, αMCA - α-muricholic acid, 7oxo-DCA - 7-oxo-deoxycholic acid, DCA - Deoxycholic acid, CA - Cholic acid, LCA - Lithocholic acid, CDCA - Chenodeoxycholic acid, UDCA - Ursodeoxycholic acid). Abbreviated names preceded with G or T indicate that the BA is conjugated with a glycine or a taurine, respectively.

## Supplementary Table 5

|  | **Bile acid** | **accurate mass** | **RT** | **ISTD, D4** | **MRM** | **ION** | **Conc,**  **CE** |
| --- | --- | --- | --- | --- | --- | --- | --- |
| 1 | Unknown T-BA_1 | 514.2838 | 3.59 | TCA-d4 | 514.3→106.9 | [CH2CHSO3]– | 84, 52 |
| 2 | Unknown T-BA_2 | 514.2838 | 4.5 | TCA-d4 | 514.3→106.9 | [CH2CHSO3]– | 84, 52 |
| 3 | Unknown T-BA_3 | 514.2838 | 4.62 | TCA-d4 | 514.3→106.9 | [CH2CHSO3]– | 84, 52 |
| 4 | Unknown T-BA_4 | 514.2838 | 4.9 | TCA-d4 | 514.3→106.9 | [CH2CHSO3]– | 84, 52 |
| 5 | Unknown T-BA_5 | 498.291 | 4.23 | TCA-d5 | 498.3→80.0 | [SO3]– | 94, 60 |
| 6 | Unknown T-BA_6 | 498.291 | 4.71 | TCA-d6 | 498.3→80.0 | [SO3]– | 94, 60 |
| 7 | Unknown T-BA_7 | 498.291 | 6.21 | TCA-d7 | 498.3→80.0 | [SO3]– | 94, 60 |
| 8 | Unknown T-BA_8 | 498.291 | 6.89 | TCA-d8 | 498.3→80.0 | [SO3]– | 94, 61 |
| 9 | Unknown T-BA_9 | 498.291 | 9.55 | TCA-d9 | 498.3→124.0 | [taurine-H]– | 90, 50 |
| 10 | Unknown G-BA_1 | 464.3009 | 4 | GCA-d4 | 464.3→74.0 | [NH2CH2COO]– | 80, 34 |
| 11 | Unknown G-BA_2 | 464.3009 | 4.54 | GCA-d4 | 464.3→74.0 | [NH2CH2COO]– | 80, 34 |
| 12 | Unknown G-BA_3 | 464.3009 | 4.73 | GCA-d5 | 464.3→74.0 | [NH2CH2COO]– | 80, 34 |
| 13 | Unknown G-BA_4 | 464.3009 | 5.06 | GCA-d6 | 464.3→74.0 | [NH2CH2COO]– | 80, 34 |
| 14 | Unknown G-BA_5 | 464.3009 | 5.19 | GCA-d7 | 464.3→74.0 | [NH2CH2COO]– | 80, 34 |
| 15 | Unknown G-BA_6 | 464.3009 | 5.37 | GCA-d8 | 464.3→74.0 | [NH2CH2COO]– | 80, 34 |
| 16 | Unknown G-BA_7 | 464.3009 | 5.48 | GCA-d9 | 464.3→74.0 | [NH2CH2COO]– | 80, 34 |
| 17 | Unknown G-BA_8 | 464.3009 | 5.65 | GCA-d4 | 464.3→74.0 | [NH2CH2COO]– | 80, 34 |
| 18 | Unknown G-BA_9 | 464.3009 | 5.98 | GCA-d4 | 464.3→74.0 | [NH2CH2COO]– | 80, 34 |
| 19 | Unknown G-BA_10 | 464.3009 | 6.22 | GCA-d5 | 464.3→74.0 | [NH2CH2COO]– | 80, 34 |
| 20 | Unknown G-BA_11 | 464.3009 | 6.59 | GCA-d4 | 464.3→74.0 | [NH2CH2COO]– | 80, 34 |
| 21 | Unknown G-BA_12 | 448.3063 | 8.62 | GUDCA-d4 | 448.3→74.0 | [NH2CH2COO]– | 52, 32 |
| 22 | Unknown G-BA_13 | 448.3063 | 8.72 | GUDCA-d4 | 448.3→74.0 | [NH2CH2COO]– | 52, 32 |

**Supplementary Table 5: Detection of unknown bile acids.**

### Supplementary figures

***
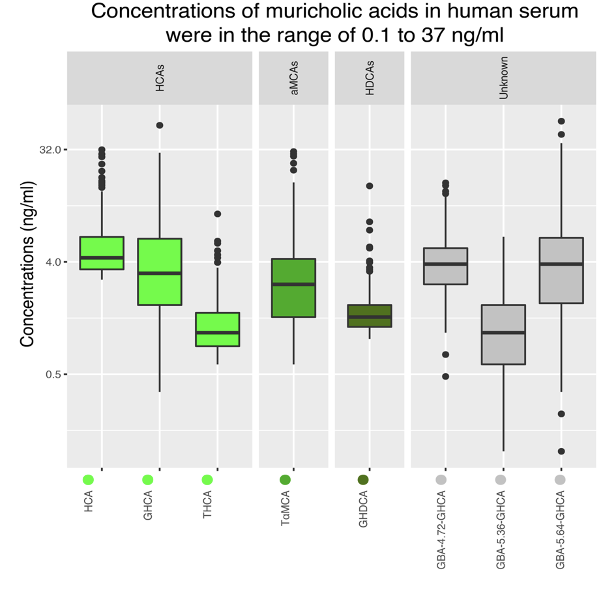
***

**Supplementary Figure 1. Fasting serum concentrations of C-6 hydroxylated bile acids in the study population of 283 individuals.** Abbreviations and complete compound name are as follows: HCA - Hyocholic acid, GHCA - glycohyocholic acid, THCA - taurocholic acid, TαMCA - tauro-α-muricholic acid, GHDCA - glycohyodeoxycholic acid.

**
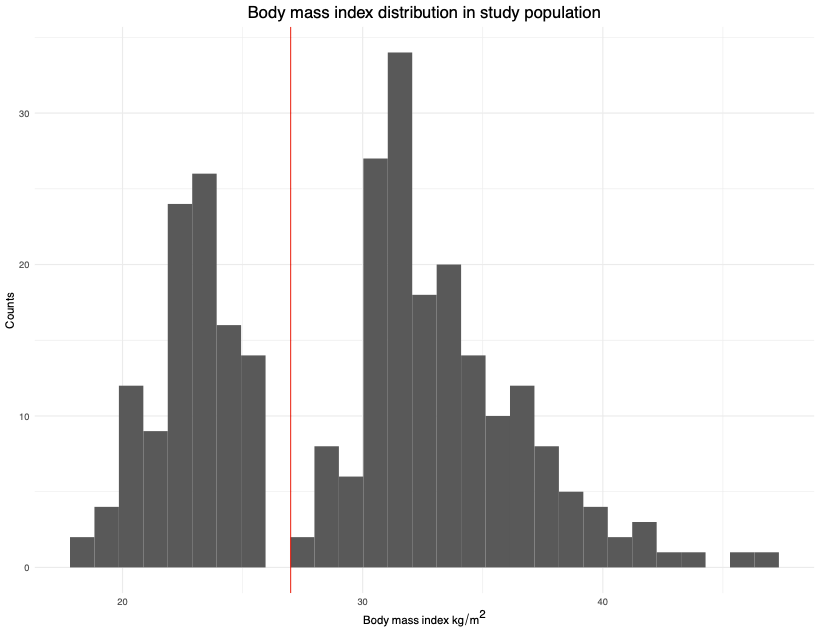
**

**Supplementary Figure 2. Body mass index (BMI) distribution in the study population of 283 individuals.** Red vertical line at BMI=27 kg/m^2^ is indicating the cut-off between non-obese and obese individuals.


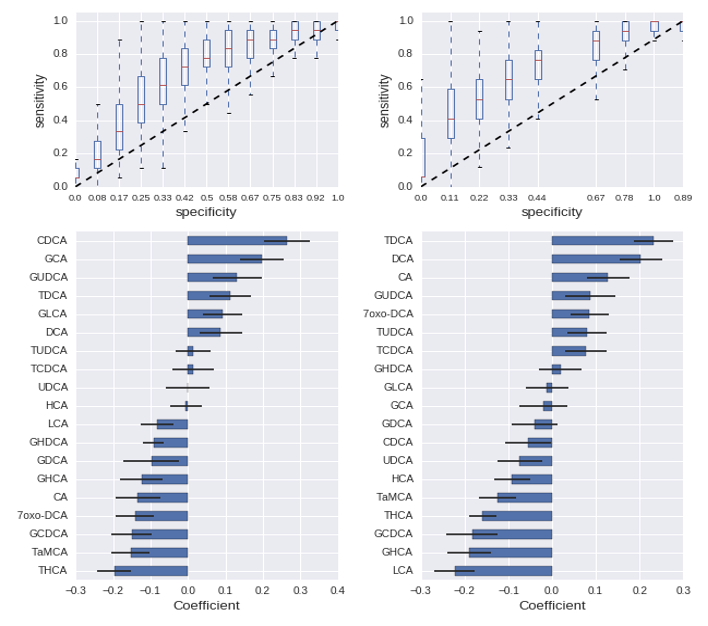


**Supplementary Figure 3. Analyses applying Logistic Ridge Regression (LR) classification models showing that the bile acids pool composition in fasting serum was different between obese and non-obese men and women, respectively, in the study population of 283 individuals.** BMI was binarized around 27 kg/m^2^ (obese versus non-obese). Upper panel: Quantile of ROC curves for men (left) and women (right) on 10,000 validation subsamples. Lower panel: Model coefficients for the obesity status classification for men (left panel) and women (right panel); the larger absolute value, the more important the given BA is distinguishing obese from non-obese individuals. Bars show the mean value of the 10,000 subsamples, with error bars indicating the standard deviation (SD). When calculating the mean and SD of model coefficients we only included estimates from the subset of the 10,000 models reaching an AUC > 0.65 (7,571 for men, 6,446 for women, respectively). Predictors were scaled so coefficients directly reflected their importance. BA abbreviations and complete compound name are: HCA - Hyocholic acid, HDCA - Hyodeoxycholic acid, αMCA - α-muricholic acid, 7oxo-DCA - 7-oxo-deoxycholic acid, DCA - Deoxycholic acid, CA - Cholic acid, LCA - Lithocholic acid, CDCA - Chenodeoxycholic acid, UDCA - Ursodeoxycholic acid). Abbreviated names preceded with G or T indicate that the BA is conjugated with a glycine or a taurine, respectively.

**
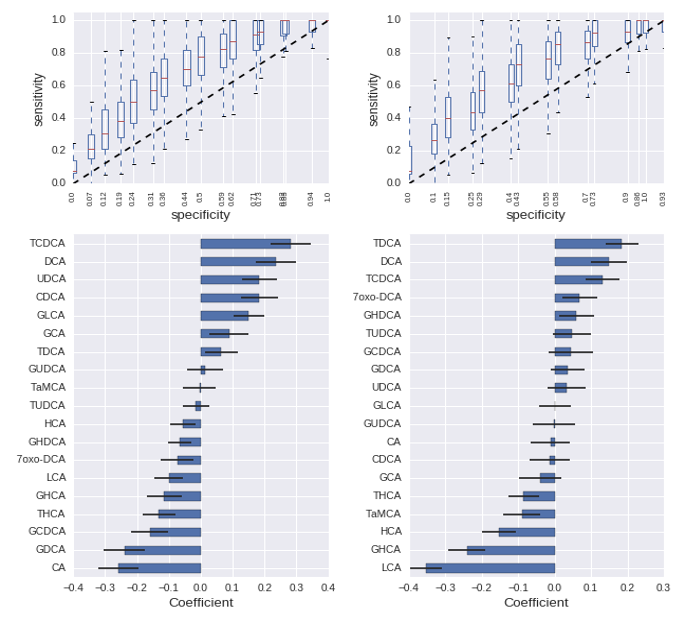
**

**Supplementary Figure 4.** **Analyses applying Logistic Ridge Regression (LR) classification models showing that the bile acids pool composition in fasting serum was different between men and women with high and low insulin resistance (HOMA-IR), respectively, in the study population of 283 individuals.** HOMA-IR was binarized around its median value. Upper panel: Quantile of ROC curves for men (left) and women (right) on 10,000 validation subsamples. Lower panel: Model coefficients for the HOMA-IR status classification for men (left panel) and women (right panel); the larger absolute value, the more important the given BA is distinguishing HOMA-IR score above the median (1.6) from individuals with HOMA-IR score below the median. Bars show the mean value of the 10,000 subsamples, with error bars indicating the standard deviation (SD). When calculating the mean and SD of model coefficients we only included estimates from the subset of the 10,000 models reaching an AUC > 0.65 (6,626 for men, 7,077 for women). Predictors were scaled so coefficients directly reflected their importance. BA abbreviations and complete compound are: HCA - Hyocholic acid, HDCA - Hyodeoxycholic acid, αMCA - α-muricholic acid, 7oxo-DCA - 7-oxo-deoxycholic acid, DCA - Deoxycholic acid, CA - Cholic acid, LCA - Lithocholic acid, CDCA - Chenodeoxycholic acid, UDCA - Ursodeoxycholic acid). Abbreviated names preceded with G or T indicate that the BA is conjugated with a glycine or a taurine, respectively.


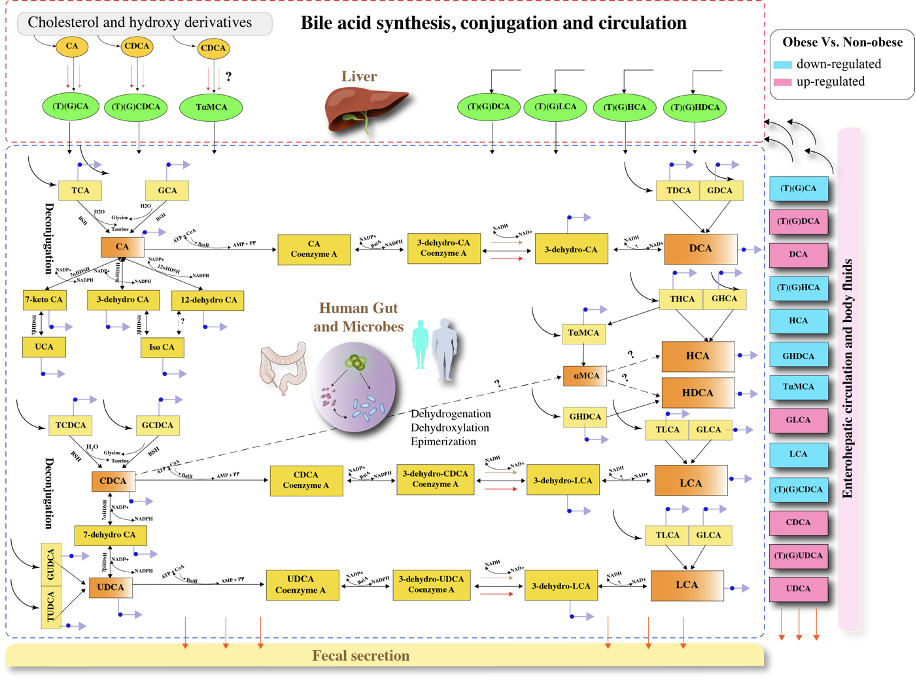


**Supplementary Figure 5.** **Modeling of the potentials of the human intestinal microbiome for de-conjugation and biotransformation of bile acids in 106 non-obese and 177 obese individuals.** In this figure, non-conjugated secondary BAs are marked with orange color, conjugated primary BAs and BA intermediates are shown in yellow in the intestinal lumen, and in green color in the liver. The regulation of serum BAs in obese versus non-obese subjects are shown by red color (up-regulated) and blue color (down-regulated). Purple arrow with blue beads denotes transport/secretion of BA to host body fluids. Unknown or putative enzymes/reactions are marked by '?'. The putative/plausible reactions are depicted by dotted lines. BA abbreviations and compound names are as follows: HCA - Hyocholic acid, HDCA - Hyodeoxycholic acid, αMCA - α-muricholic acid, 7oxo-DCA - 7-oxo-deoxycholic acid, DCA - Deoxycholic acid, CA - Cholic acid, LCA - Lithocholic acid, CDCA - Chenodeoxycholic acid, UDCA - Ursodeoxycholic acid. Abbreviated names preceded with G or T indicate that the BA is conjugated with glycine or taurine, respectively.


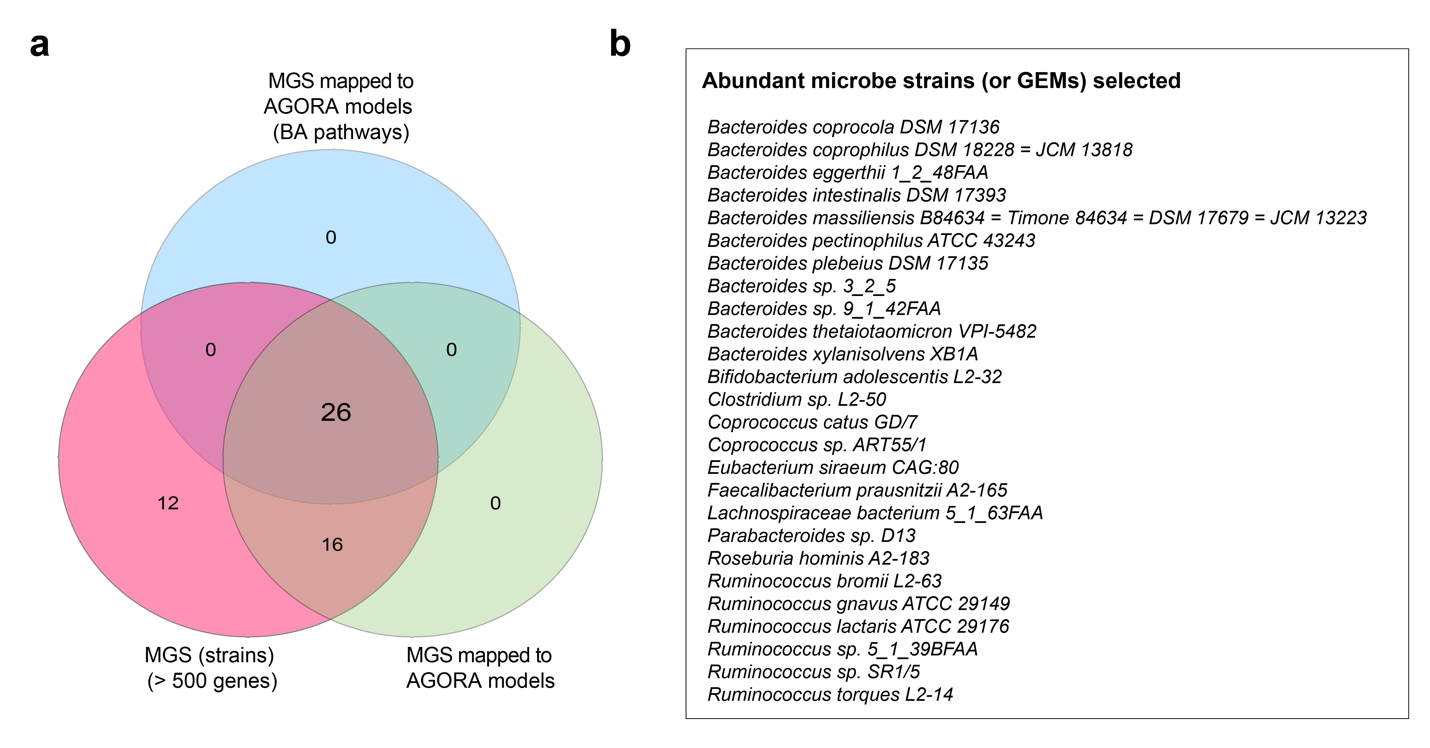


**Supplementary Figure 6.** **Selection of abundant gut bacterial species/strains in the studied 283 individuals and illustration of their corresponding genome-scale models (GEMs) according to the AGORA compendium**. A) Venn diagram comparing metagenomic species (MGS >500 genes) in this study, GEMs from AGORA compendium, and GEMs enhanced with bile acids (BA) biotransformation pathways from 'AGORA_BA' compendium and Virtual Metabolic Human database. B) Bacterial strains and their corresponding GEMs employed in this study.


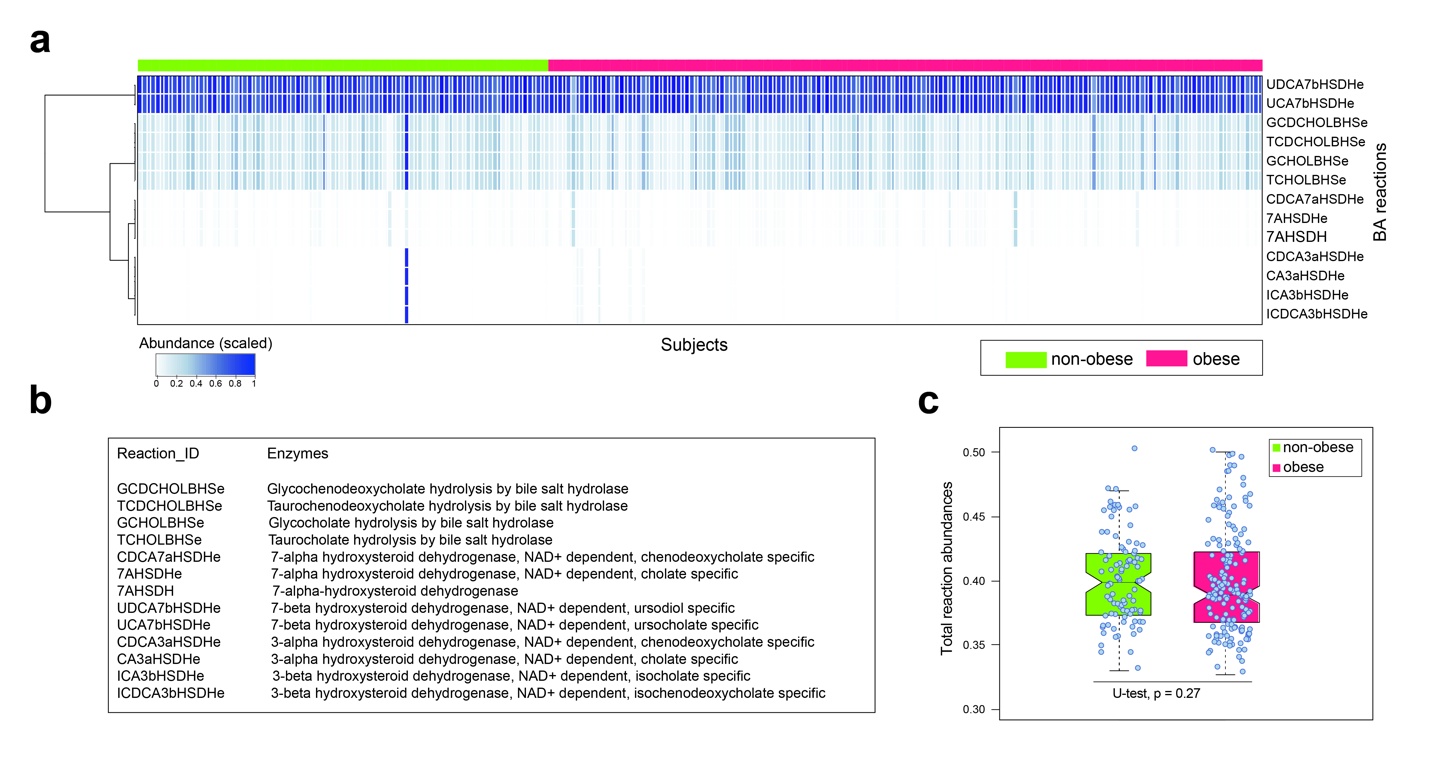


**Supplementary Figure 7.** **Reaction abundances (RAs) of** **bile acids (BAs) in 106 non-obese and 177 obese individuals**. A) Abundances of the BA reactions in non-obese and obese individuals inferred from 26 'AGORA_BA' models. B) Description of BAs reactions as shown in panel 'A'. Each RA was normalized by the maximum abundance in the community. C) Total RA in non-obese and obese subjects evaluated by Mann-Whitney U test.


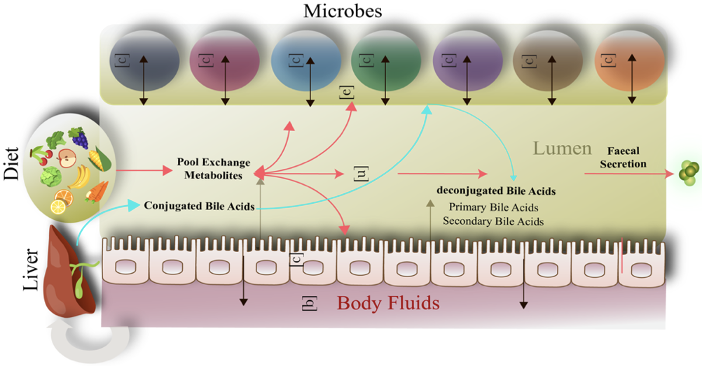


**Supplementary Figure 8. Illustration of the applied multi-compartment Genome-Scale Metabolic Modeling framework**. It comprised of 26 selected "AGORA_BA" models with each model having a cytoplasm [c] and an extracellular space [e] for exchange of metabolites. A common lumen compartment [u] that allowed exchange of pooled metabolites between bacteria and gut epithelial cells is shown. The luminal compartment [u] accommodated dietary fluxes (mm/g dry weight/hr) and faecal secretion. In addition, the lumen could take up and secrete conjugated and non-conjugated BA, respectively, when the bacterial strain and corresponding enzyme(s) were known. The framework was coupled with a host epithelial cell model (*hLC_001*), reconstructed in this study. A compartment simulating host body fluids [b] is also shown.


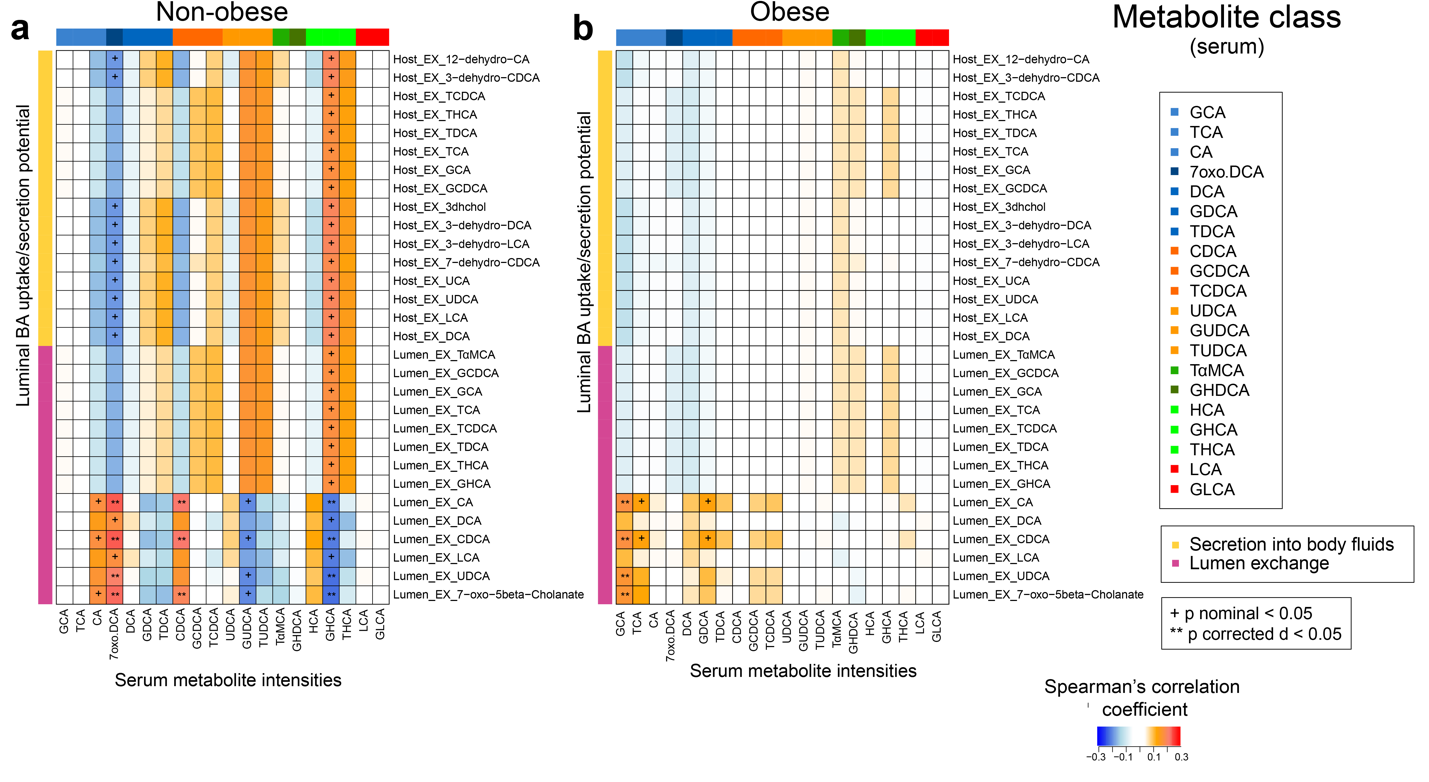


**Supplementary Figure 9.** **Spearman's rank-based correlation analyses between modeled uptake and/or secretion potentials of bile acids in the intestinal lumen and the measured fasting serum BA levels in 106 non-obese and 177 obese individuals**. A-B) Correlation between estimated luminal BA exchange potential (uptake/lUP and/or secretion/lSP) and the measured serum BA levels of the non-obese subjects and obese subjects, respectively. Orange and blue color represents positive and negative value of correlation coefficients, respectively. White denotes no correlation, ‘+’ denotes nominal (p-values < 0.05) and ‘**’ denotes FDR adjusted (p-values < 0.05). Prefix *‘Host_EX’* represents the BA secretion potential into the body fluids. BA abbreviations and compound names are: HCA - Hyocholic acid, HDCA - Hyodeoxycholic acid, αMCA - α-muricholic acid, 7oxo-DCA - 7-oxo-deoxycholic acid, DCA - Deoxycholic acid, CA - Cholic acid, LCA - Lithocholic acid, CDCA - Chenodeoxycholic acid, UDCA - Ursodeoxycholic acid. Abbreviated names preceded with G or T indicate that the BA is conjugated with glycine or taurine respectively.


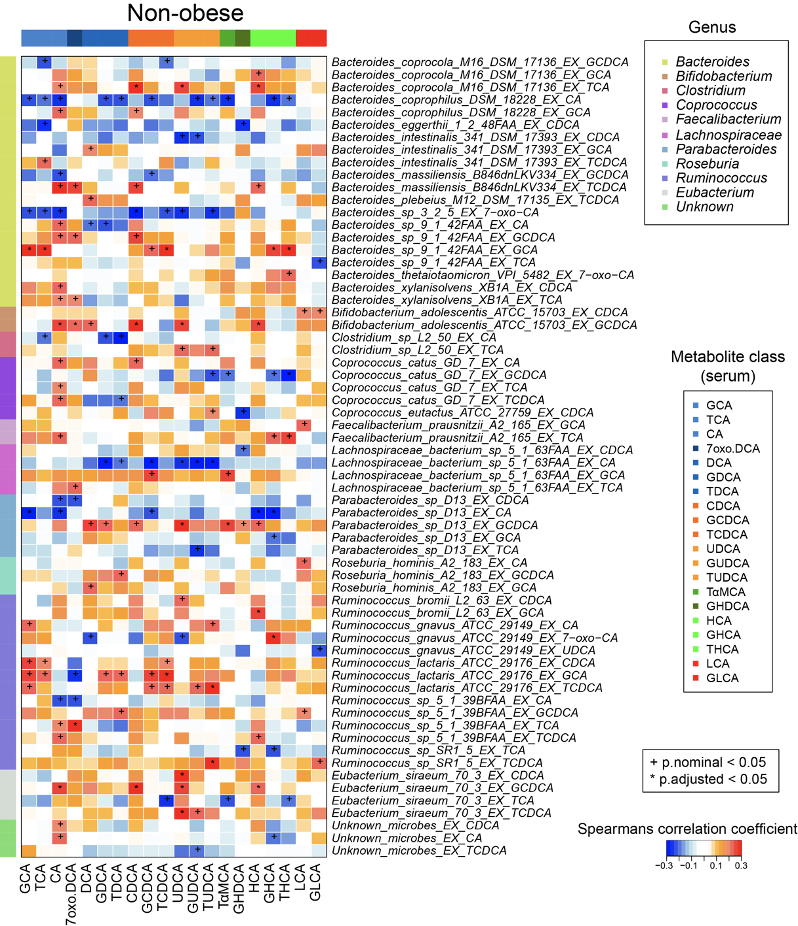


**Supplementary Figure 10.** **Spearman's rank-based correlation analyses between estimated metabolic potential of gut bacteria and measured serum bile acids in 106 non-obese individuals**. Red/orange and blue color represents positive and negative value of correlation coefficients, respectively. White represents no correlation, ‘+’ denotes nominal p-values < 0.05 and ‘*’ denotes (FDR corrected p < 0.05). Each BA reaction is suffixed with the associated bacterial strain. The circulating BAs are color coded by their chemical classes. BA abbreviations and complete compound name are: HCA - Hyocholic acid, HDCA - Hyodeoxycholic acid, αMCA - α-muricholic acid, 7oxo-DCA - 7-oxo-deoxycholic acid, DCA - Deoxycholic acid, CA - Cholic acid, LCA - Lithocholic acid, CDCA - Chenodeoxycholic acid, UDCA - Ursodeoxycholic acid). Abbreviated names preceded with G or T indicate that the BA is conjugated with a glycine or a taurine, respectively.

***
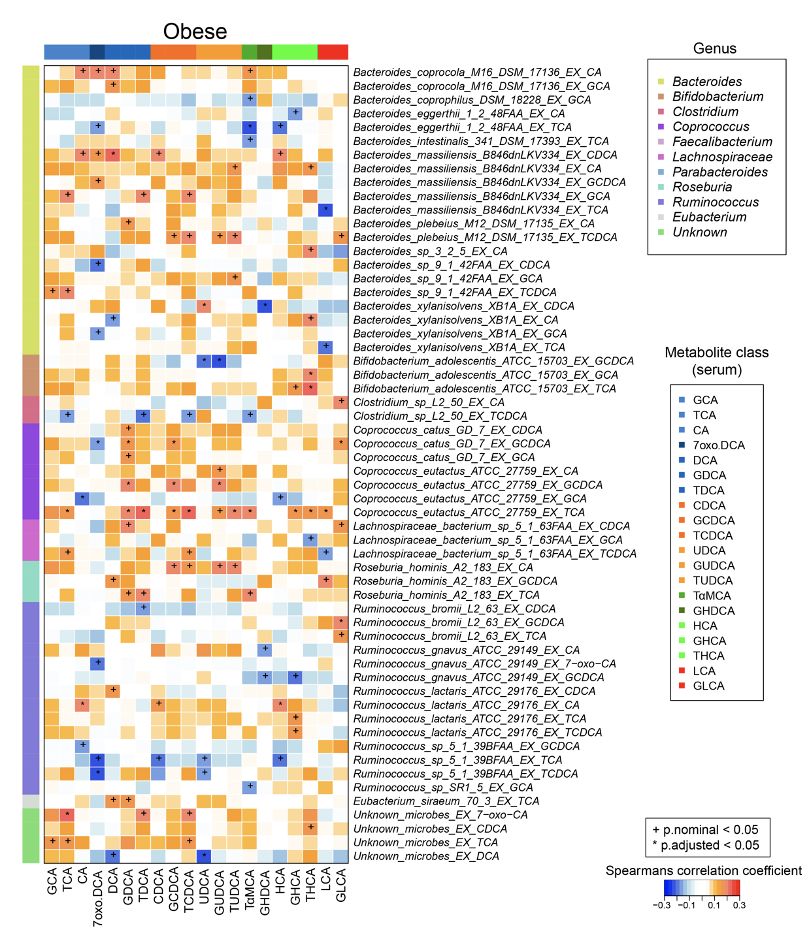
***

**Supplementary Figure 11. Spearman's rank-based correlation analyses between estimated metabolic potentials of gut bacteria and measured serum bile acids in 177 obese individuals**. Red/orange and blue color represents positive and negative value of correlation coefficients, respectively. White represents no correlation, ‘+’ denotes nominal p-values < 0.05 and ‘*’ denotes (FDR corrected p < 0.05). Each BA reaction is suffixed with the associated bacterial strain. The circulating BAs are color coded by their chemical classes. BA abbreviations and complete compound name are as follows: HCA - Hyocholic acid, HDCA - Hyodeoxycholic acid, αMCA - α-muricholic acid, 7oxo-DCA - 7-oxo-deoxycholic acid, DCA - Deoxycholic acid, CA - Cholic acid, LCA - Lithocholic acid, CDCA - Chenodeoxycholic acid, UDCA - Ursodeoxycholic acid). Abbreviated names preceded with G or T indicate that the BA is conjugated with a glycine or a taurine, respectively.

***
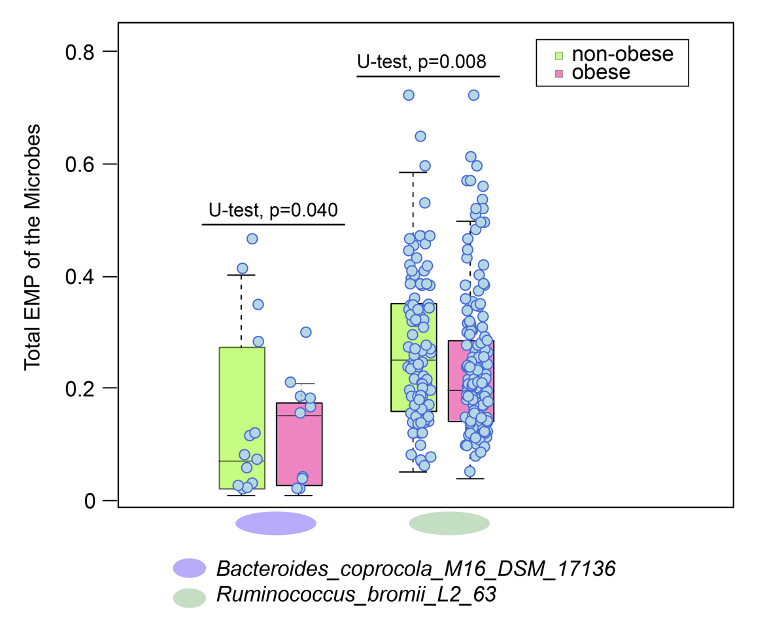
***

**Supplementary Figure 12**. **Intestinal bacterial strains that in modeling analyses showed significant differences in the total estimated metabolic potentials) in 106 non-obese and 177 obese individuals.** Box plot represents significant difference in the total EMP between non-obese and obese subjects as evaluated by Mann-Whitney U test.

***
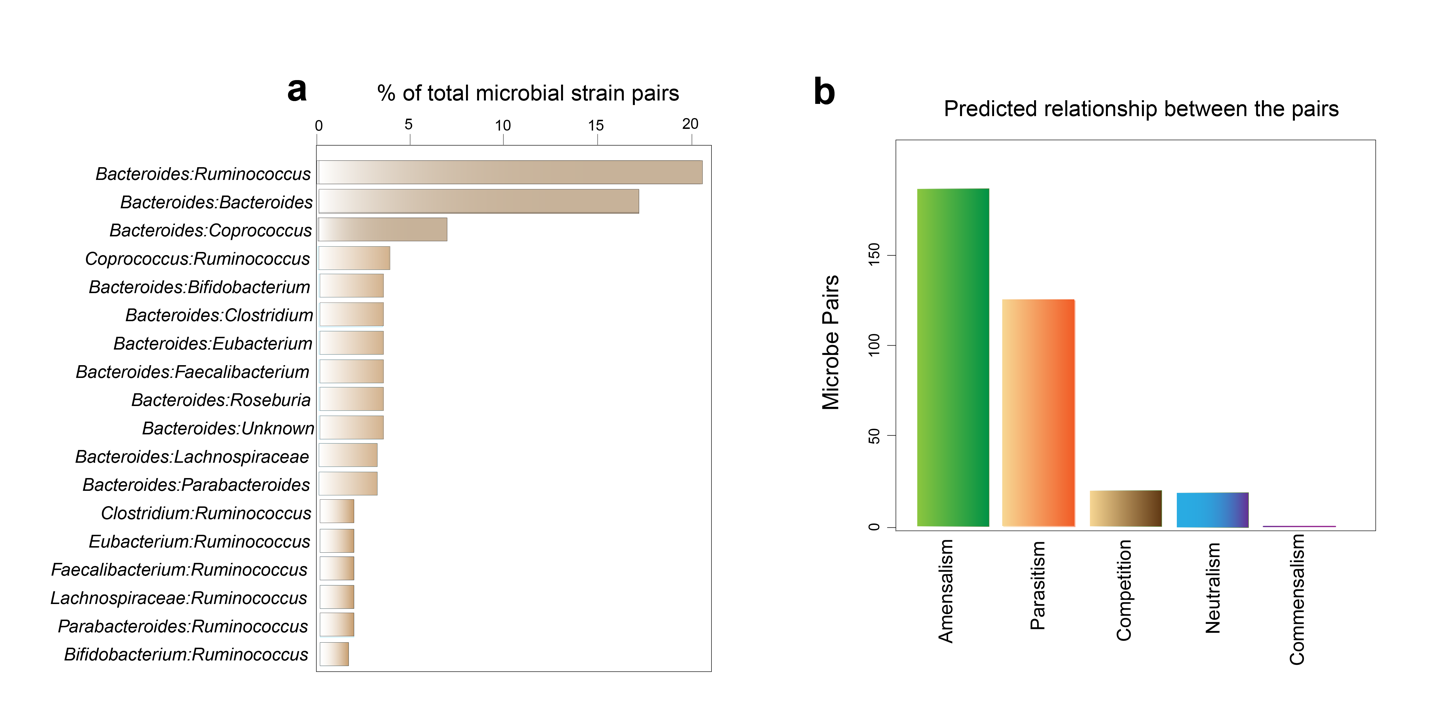
***

**Supplementary Figure 13.** **Modeling of pairwise interactions between 26 selected gut bacterial strains (*co-growth*) subjected to Average European Diet (AED)**. A) Gut bacterial strain pairs that can biotransform BAs were grouped by their genus. Genus with at least two or more strain pairs (>1% of total strain pairs) are shown. B) The predicted relationship between all possible pairs of examined bacteria engaged in BA de-conjugation and BA biotransformation. *Amensalism*: In the paired simulation, one microbe grows slower than the other does, but their growth is not mutually dependent. *Parasitism*: One microbe grows faster than another does. *Competition*: In the paired simulation, both microbes exhibit slower growth as compared with their individual growth. *Neutralism*: Independent and unaffected growth of the microbes in the paired simulations. *Commensalism*: Growth of one microbe is faster and unaffected by the other. The definitions of mentioned concepts are derived from Magnúsdóttir, S. et al^2^.

**REFERENCES**

1. Qin, N. et al. Alterations of the human gut microbiome in liver cirrhosis. *Nature* **513**, 59 (2014).

2. Magnúsdóttir, S. et al. Generation of genome-scale metabolic reconstructions for 773 members of the human gut microbiota. *Nat. Biotechnol.* **35**, 81 (2017).

3. Noronha, A. et al. The Virtual Metabolic Human database: integrating human and gut microbiome metabolism with nutrition and disease. *Nucleic Acids Res.* **47**, D614-D624 (2018).

4. Heirendt, L. et al. Creation and analysis of biochemical constraint-based models: the COBRA Toolbox v3. 0. *arXiv preprint arXiv:1710.04038* (2017).

5. Baldini, F. et al. The Microbiome Modeling Toolbox: from microbial interactions to personalized microbial communities. *Bioinformatics* **35**, 2332-2334 (2018).

6. Gudmundsson, S. & Thiele, I. Computationally efficient flux variability analysis. *BMC Bioinformatics* **11**, 489 (2010).

7. Brunk, E. et al. Recon3D enables a three-dimensional view of gene variation in human metabolism. *Nat. Biotechnol.* **36**, 272 (2018).

8. Sahoo, S. & Thiele, I. Predicting the impact of diet and enzymopathies on human small intestinal epithelial cells. *Hum. Mol. Genet.* **22**, 2705-2722 (2013).

9. Watanabe, M., Fukiya, S. & Yokota, A. Comprehensive evaluation of the bactericidal activities of free bile acids in the large intestine of humans and rodents. *J. Lipid Res.* **58**, 1143-1152 (2017).

10. Heinken, A. et al. Personalized modeling of the human gut microbiome reveals distinct bile acid deconjugation and biotransformation potential in healthy and IBD individuals. *BioRxiv*, 229138 (2017).

11. Swann, J.R. et al. Systemic gut microbial modulation of bile acid metabolism in host tissue compartments. *Proc. Natl. Acad. Sci. U. S. A.* **108**, 4523-4530 (2011).

12. Kumar, M. et al. Gut microbiota dysbiosis is associated with malnutrition and reduced plasma amino acid levels: lessons from genome-scale metabolic modeling. *Metab. Eng.* **49**, 128-142 (2018).

13. Lazaridis, K.N., Gores, G.J. & Lindor, K.D. Ursodeoxycholic acid ‘mechanisms of action and clinical use in hepatobiliary disorders’. *J. Hepatol.* **35**, 134-146 (2001).

14. Wahlström, A., Sayin, S.I., Marschall, H.-U. & Bäckhed, F. Intestinal crosstalk between bile acids and microbiota and its impact on host metabolism. *Cell Metab.* **24**, 41-50 (2016).

15. Ridlon, J.M., Kang, D.-J. & Hylemon, P.B. Bile salt biotransformations by human intestinal bacteria. *J. Lipid Res.* **47**, 241-259 (2006).

16. Doden, H. et al. Metabolism of oxo-bile acids and characterization of recombinant 12α-hydroxysteroid dehydrogenases from bile acid 7α-dehydroxylating human gut bacteria. *Appl. Environ. Microbiol.* **84** (2018).

17. Jeffery, I.B., Claesson, M.J., O'toole, P.W. & Shanahan, F. Categorization of the gut microbiota: enterotypes or gradients? *Nat. Rev. Microbiol.* **10**, 591 (2012).

18. Knights, D. et al. Rethinking “enterotypes”. *Cell Host Microbe* **16**, 433-437 (2014).

19. Dao, M.C. et al. Akkermansia muciniphila and improved metabolic health during a dietary intervention in obesity: relationship with gut microbiome richness and ecology. *Gut* **65**, 426-436 (2016).

20. Miquel, S. et al. Ecology and metabolism of the beneficial intestinal commensal bacterium Faecalibacterium prausnitzii. *Gut Microbes* **5**, 146-151 (2014).

21. Friedman, J., Hastie, T. & Tibshirani, R. The elements of statistical learning, Vol. 1. (Springer series in statistics New York, 2001).

22. Jäntti, S.E. et al. Quantitative profiling of bile acids in blood, adipose tissue, intestine, and gall bladder samples using ultra high performance liquid chromatography-tandem mass spectrometry. *Anal. Bioanal. Chem.* **406**, 7799-7815 (2014).
